# Novel formulation of c-di-GMP with cytidinyl/cationic lipid reverses T cell exhaustion and activates stronger anti-tumor immunity

**DOI:** 10.7150/thno.71010

**Published:** 2022-09-11

**Authors:** Xiaotong Yu, Jing Yu, Hong Dai, Chenyun Deng, Xudong Sun, Sijie Long, Zhujun Jiang, Hongyan Jin, Zhu Guan, Zhenjun Yang

**Affiliations:** 1 State Key Laboratory of Natural & Biomimetic Drugs, School of Pharmaceutical Sciences, Peking University, No.38 Xueyuan Road, Haidian District, Beijing 100191, China.; 2 Department of Obstetrics and Gynecology, Peking University First Hospital, No. 8 Xishiku Street, 100034, Beijing, China.

**Keywords:** c-di-GMP, DNCA, CLD, T cell exhaustion, STING, immunotherapy

## Abstract

**Rationale:** Cyclic dinucleotides (cDNs) are a promising class of immunotherapeutic agent targeting stimulator of interferon genes (STING). However, enzymatic instability and transmembrane barriers limit the extensive clinical application of cDNs. Thus, a novel delivery system, composed of a neutral cytidinyl lipid DNCA and a cationic lipid CLD (Mix) that interacts with cDNs via H-bonding, pi-stacking and electrostatic interaction, is developed and optimized to overcome the above issues.

**Methods:** The optimal composition of Mix for cDNs encapsulation was explored with RAW-Lucia ISG cells. The physicochemical properties of resulted nanoparticles were characterized. To validate the anti-tumor immunity of cDNs/Mix both *in vitro* and *in vivo*, immunogenic cell death (ICD) related markers and tumor inhibition efficacy were evaluated in cancer cells and tumor models, respectively. The mechanism by which cdG/Mix exerted the antitumor effects was explored by flow cytometric analysis and *in vivo* depletion.

**Results:** Based on our developed and optimized delivery system, neutral cytidinyl lipid DNCA/cationic lipid CLD (Mix), cdG (500 nM *in vitro*, 1-10 μg *in vivo*)/Mix not only more potently stimulated production of IFNβ and related cytokines including CXCL9 and CXCL10, promoted ICD, led to NK and CD8^+^ T cell activation, inhibited tumor growth in both EO771 and B16F10 models and increased their survival rate (~43%), but also obviously reversed the T cell exhaustion (Tex) in tumor, meanwhile down regulated the mRNA expression of *Tox* and *Nr4a*, which are key regulators of Tex.

**Conclusion:** cdG/Mix triggered ICD in various cancer cells and reversed the Tex systemically in tumor-burden mice, which would be a promising alternative strategy for cancer immunotherapy.

## Introduction

The activation of the cGAS-STING pathway has tremendous potential to improve anti-tumor immunity 1. Chromosome instability, DNA damage, oncosuppressor gene mutation and deletion in cancer cells lead to the cytoplasmic DNA accumulation, which is recognized by cyclic-GMP-AMP synthase and then leads to production of 2ʹ,3ʹ-cyclic guanosine monophosphate-adenosine monophosphate (2ʹ,3ʹ-cGAMP) 1. Cyclic diguanylate (c-di-GMP, cdG), as well as 3ʹ,3ʹ-cGAMP, and c-di-AMP produced by bacteria, and 2ʹ,3ʹ-cGAMP from mammals, are novel immunotherapeutics that can activate the stimulator of interferon genes (STING), a pattern recognition receptor that localizes to the endoplasmic reticulum, and induce potent type I interferon (IFN-Ⅰ) response, leading to the accumulation and infiltration of CD8^+^ T cells in tumors 1.

(Rp,Rp)Dithio-cyclic[A(2′,5′)pA(3′,5′)p] (ADU-S100) is a candidate in clinical trials (NCT02675439) which profoundly inhibits tumor growth by intratumoral administration (i.t., 50 μg) in CT26 or 4T1 tumor models, however, shows limited clinical activity in patients with advanced/metastatic solid tumors or lymphomas (50-6400 μg) 2. Small molecule MSA-2 is a potential non-cyclic dinucleotide STING agonist in pre-clinical stage, which significantly inhibits tumor growth and achieves long-term survival rates of 35% and 100% in MC38 mice model with dosage administered orally (80 mg/kg) or subcutaneously (50 mg/kg) 3. However, intense STING activation leads to T cell apoptosis and limits the T cell response with higher dosage 4-6.

Recent reports suggest some carriers, including liposomes, polymers, peptides, and inorganic nanoparticles, can reduce the curative dosage of cyclic dinucleotides (cDNs) to below 20 μg in monotherapy 7-19. Encapsulated cDNs can promote the polarization of M1 macrophages, dendric cell priming, and antitumor NK and T cell activation and infiltration in tumor 16,19,20. Furthermore, encapsulated 2',3'-cGAMP (800 nM) potently promotes cancer immunogenic cell death (ICD) in neuroblastoma, which is a promising strategy to enhance the immunogenicity of tumor and trigger the inhibited immune system to kill tumor cells 21. All of the above effects of cDNs (0.1-20 μg) enhance the anti-tumor immunity and achieve effective tumor suppression with 10-60% long term-survival rate in tumor models 7-19,22,23. However, the reported cationic lipid derived delivery systems induced different degrees systematic toxicity, including blood incompatibility, immunogenicity 24-27.

A novel delivery system (Mix) developed by our group consists of neutral cytidinyl lipid (DNCA) and cationic lipid (CLD), which can interact with nucleic acid drugs via H-bonding, pi-stacking and electrostatic interaction, and facilitate the nanocomplex formation with appreciable size and zeta potentials 28,29. By local, intravenous or intramuscular injections, Mix can deliver aptamer, antisense oligonucleotide, siRNA and mRNA with remarkable therapeutic effect and safety in vivo 28,30-35. Further optimization of the delivery system, cDNs/Mix have displayed similar immunostimulatory activity and anti-tumor efficacy to other carriers at lower dosage (1 μg, i.t.), as well as ICD induction in melanoma and breast cancer cells (0.5-1.4 μM). Notably, cdG/Mix can reverse the T cell exhaustion (Tex) both in tumor and systemic immune organization, meanwhile down-regulate the mRNA expression of *Tox* and *Nr4a* 36.

## Materials and Methods

### Synthesis of cdG, 2ʹ,3ʹ-cGAMP and 3ʹ,3ʹ-cGAMP

**Scheme 1:**
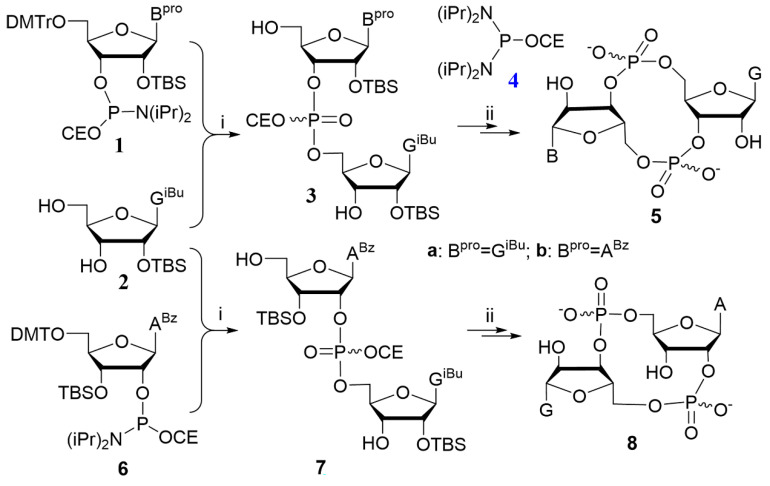
The reported one-pot phosphoramidite method for the synthesis of cyclic dinucleotides optimized by our group. Reagents and conditions: i. (a) 1*H*-tetrazole, anhyhrous CH_3_CN, rt, 5 h; (b) TBHP (5-6 M in decane), rt, 10 min; (c) CF_3_COOH, CH_2_Cl_2_, rt, 20 min; ii. (a) **4**, 1*H*-tetrazole, anhyhrous CH_3_CN, rt, 5 h; (b) TBHP (5-6 M in decane), rt, 10 min; (c) CH_3_NH_2_ in MeOH, rt, 3 h; (d) Et_3_N•3HF, pyridine, 50 °C, 1 h.

The one-pot phosphoramidite method was adopted to synthesize cdG etc., which was introduced before 37,38. General procedures for synthesis are provided in Supplementary Materials (**Scheme 1**). ^1^H NMR, ^31^P NMR and HRMS spectrums of cdG etc. used in this study are provided in the Supplementary Materials (**Figure S10-18**).

### Nanoparticle preparation

The DNCA and CLD liposome were dissolved in ethanol for stock solutions (2.5 mM). The cdG were dissolved in dH_2_O at 125 μM. The PEG2000-DSPE were dissolved in ethanol at 20 μM. Subsequently, 0.4 nmol cdG (3.2 μL), 4 nmol DNCA (1.6 μL), 2.4 nmol CLD (0.96 μL), and 0.032 nmol DSPE-PEG2000 (1.6 μL) was diluted with 72.6 μL PBS (M&C, Beijing, China), or GenOpti (M&C). Afterwards, ultrasonic process the above solution for 30 minutes at 26 °C. As a result, nanocomplexes were obtained (cdG 5 μM, 80 μL) for usage. Entranster^TM^-*in vivo* was purchased from Engreen Biosystem Co, Ltd. And cdG-Entranster was prepared according to the manufacturer's instructions. cdG-NTL was prepared according to the reference 39.

### Characterization of nanoparticle

The hydrodynamic diameter and zeta potential were characterized by Malvern Zetasizer Nano ZS90 instrument with photon correlation spectroscopy at a scattering angle of 90°. Data were analyzed using an ELS-8000 software package. Lipid morphologies were imaged on a 120 kV JEM-1400Plus Transmission Electron Microscope with 10 μL samples (at 0.3 mg/mL).

*In vitro* drug release study was performed as follows: transfer 2 ml of the cdG/Mix(168 μM) into a dialysis bag (Spectrumlabs; 2 kDa molecular weight cut-off), clamp the two sections of the dialysis bag, and then completely immersed into 36 mL PBS. Place the whole device on a magnetic heating stirrer (37 °C, 500 rpm), and 5 μL of buffer was taken at each scheduled time point and quantified using a Thermo NanoDrop 2000C spectrophotometer. The cumulative release rate was calculated according to equation (1), the release was analyzed using first order (2) and Higuchi (3) models.

Cumulative release rate (%) = amount of released cdG in buffer solution/amount of cdG loaded initially × 100% (1)

ln(1 - M_t_ / M_∞_) = - k_1_ t (2)

M_t_ / M_∞_ = k_H_ t^1/2^ (3)

Where*,* 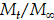 is the fraction of drug release, *k_1_* and *k_H_* are first order rate constant and Higuchi drug release constant.

### Cell culture

The B16F10 and EO771 cells were kindly provided by Pro. Ning Tao (Institute of biophysics, Chinese academy of sciences) and cultured in Roswell Park Memorial Institute (RPMI) 1640 medium (M&C) supplemented with 10% fetal bovine serum (FBS, Gibco). RAW264.7 and THP-1 cells (cell bank of the Committee on Type Culture Collection of Chinese Academy of Science) were cultured in DMEM medium (M&C) and RPMI-1640 medium (M&C), respectively, supplemented with 10% FBS. RAW-Lucia ISG and THP1-Dual ISG cells were kindly provided by Pro. Changpo Chen in Henan Normal University, China and cultured according to the manufacturer's instructions. No authentication of the cell lines was performed by the authors. All cell lines were grown in a humidified atmosphere with 5% CO_2_ at 37 °C and used before passage 10 after purchase from the vendor.

### Induction of type I interferon in RAW-Lucia ISG Cells and THP1-Dual ISG cells

RAW-Lucia ISG cells and THP1-Dual ISG cells were used to determine IRF pathway activation by the indicated formulations. Cells (180 μL) were inoculated in 96 well plates (100,000 cells/well) with DMEM and RPMI-1640 medium, respectively, and incubated for 24 h at 37 °C in 5% CO_2_. Then, cells were stimulated for 18 h at 37 °C in a 5% CO_2_ incubator with 20 μL of various cDNs-containing formulations. The quantity of type I interferon was indirectly quantified using QUANTI-Luc (InvivoGen), which was prepared and used according to the manufacturer's instructions.

### Flow cytometric analysis of cellular uptake

RAW264.7 cells were seeded in 12-well plates at 300,000 cells well^-1^ 1 day before transfection and treated with indicated cdGMP-Dy547 (Axxora) formulations for 2 h at concentrations of 500 nM. Subsequently, the cells were harvested and centrifuged at 1000 g for 3 min, and precipitates were washed by pre-cooled PBS and filtered for a homogeneous distribution in the solution. The cellular cDNs uptake was analyzed on a BD LSRFortessa with 561 nm excitation laser and 582/15 filter configuration.

### Flow cytometric analysis of apoptosis and calreticulin

Cell apoptosis was detected by Flow cytometry using FITC Annexin V Apoptosis Detection Kit I (556547, BD Pharmingen) according to the manufacturer's instructions. Cells (2×10^5^ cells/well in a 12-well plate) were treated with PBS, Mix, free cdG, or cdG/Mix for 24 hours. Cells were washed twice with PBS and then resuspended in 1× Binding Buffer. Cells were stained with Annexin V and PI for 15 mins at RT in the dark, and run on the CytoFLEX S flow cytometer (BECHMAN COULTER). Data from three independent experiments were analyzed using CytExpert software.

Calreticulin (CRT) expression was detected by Flow cytometry using Alexa Fluor 647-conjugated anti-CRT antibody (Ab196159, Abcam). Cells (2×10^5^ cells/well in a 12-well plate) were treated with PBS, Mix, free cdG, or cdG/Mix for 24 hours. cells were trypsinized, washed in cold PBS, and stained with anti-CRT antibody for 1 h at 4 °C, and stained with PI for 5 mins at RT in the dark then washed three times and re-suspended in cold PBS with 3% FBS and analyzed on a CytoFLEX S flow cytometer (BECHMAN COULTER). Data from three independent experiments were analyzed using CytExpert software.

### ATP detection assay

The release of ATP was measured with the ATP Determination Kit (A22066, Molecular Probes). Prepare the reaction solution according to the protocol, mix the cell supernatant or ATP standard after administration with the reaction solution, and luminescence was measured by the LB960 Microplate Luminescence Detector (Berthold). Luminescence was normalized to background from untreated blank group. Results were from three independent experiments.

### Confocal microscopy assay

RAW264.7 cells (2 × 10^4^ per well) were cultured at confocal observation dishes and proliferated for 24 h. Then, cdG-Dy547 (500 nM) were exposed to cells and incubated for 1, 2 and 4 h. Subsequently, the culture medium was removed, following by washing twice with PBS. Cells were stained with Hoechst 33342 (Solarbio) for 15 min and washed twice with PBS, and then observed under an A1Rsi confocal microscope (Nikon Instruments Inc). Confocal images were obtained using NIS-Elements software (Nikon Instruments Inc).

### Mouse care and experimentation

Female C57BL/6 mice (6-8 weeks old) were purchased from Vital River Laboratory Animal Center (Beijing, China). All animals were maintained at the animal facilities of Peking University under specific pathogen-free conditions and treated in accordance with the regulations and guidelines of the Institutional Animal Care and Use Committee.

### Gene expression analysis following intratumoral administration

Female C57BL/6 mice (6-8 weeks old) were inoculated subcutaneously with 200,000 B16F10 cells. Mice were treated intratumorally with PBS, free cdG (5 μg) or cDNs (5 μg)/Mix when tumors were around 200 mm^3^. For qPCR analysis, tumors were collected on indicated time points, then grinded and lysed immediately using a TRIzol reagent (Invitrogen) according to the standard extraction protocol. For cDNA synthesis, total RNA (1 μg) was reverse-transcribed using the Reverse Transcription System (Promega). Then, the real-time PCR was carried out using an Mx3005P QPCR System (Agilent) and SYBR Green QPCR Master Mix (Promega). The expression levels were determined by the Ct (cycle threshold) values and analyzed using delta-delta-Ct method. The primers used in the study were listed in Table S1.

### Murine tumor experiments

B16F10 cells (200,000) were injected subcutaneously into the flank of 6-8-week-old female C57BL/6 mice suspended in 100 μL of serum-free RPMI 1640 media. Five cdG/Mix etc. injections were given as treatment scheme when tumors grew to between 50-100 mm^3^. E0771 cells (750,000) were injected subcutaneously into the flank of 6-8week-old female C57BL/6 mice in 100 μL of serum-free RPMI 1640 media. When tumors grew to between 50-100 mm^3^, cdG/Mix were initially administered by either i.v. or i.t*.* according to the treatment scheme. The tumor size was measured using caliper, and the tumor volume calculated using the equation V=1/2×L×W^2^ 40.

### Flow cytometric analysis in EO771 mice

All tissue preparations were performed after Murine euthanasia. Peripheral blood was collected via mice eye, then transferred into sodium heparin-coated vacuum tubes before dilution in PBS with 5 mM ethylenediaminetetraacetic acid (EDTA, 60-00-4, Aladdin) and 0.5% bovine serum albumin (BSA, 554657, BD Pharmingen). Spleens and lymph nodes were crushed using a sterile syringe piston and screened with a 70 μm cell filter in PBS with 1 mM EDTA and 2% FBS at 4 °C. Cells were centrifuged at 300 g for 10 min at 4 °C and re-suspended in PBS. Tumors were finely minced and digested in RPMI 1640 with Mouse tumor tissue dissociation kit (Miltenyi, 130-096-730), then inverted in the tissue dissociator to run program as the protocol. Cell suspensions were screened with a 70 μm cell filter. After counting, all cells were diluted into 1×10^6^ cells/ml cell suspension for later use. Each 100 ul cell suspension was preincubated with 2 ul CD16/CD32 antibodies at 4 °C for 5 min to block Fc receptors 41.

Dead cells were excluded by staining with Live/Dead fixable viability stain 510 (564406, BD Biosciences). Cell surface staining was performed in brilliant stain buffer (563794, BD Horizon) for 30 min at 4 °C in dark. Intracellular staining (CD206 and Granzyme B) was performed with Transcription Factor Buffer Set RUO (562574, BD Pharmingen) after fixing cells with Fix/Perm buffer for 20 min at 4 °C in dark and permeabilizing cells with Intracellular Staining Perm/Wash Buffer. The following anti-mouse antibodies were used: (PE-Cy5)-CD3 (clone BM10-37), (FITC)-CD4 (clone V4), (APC-Cy7)-CD8 (clone 53-6.7), (BUV395)-CD45 (clone 30-F11), (PerCP-Cy5.5)-CD38 (clone 90), (Alexa Fluor 647)-CD101 (clone 307707), (PE-Cy7)-CD86 (clone GL1), (PE-CF594)-CD80 (clone 16-10A1), (PerCP-Cy5.5)-MHC class II (clone M5/114.15.2), (BV605)-CD11c (clone HL3), (BV650)-CD11b (clone M1/70), (PE)-F4/80 (clone T45-2342), (Alexa Fluor 700)-NK-1.1 (clone PK136), (Alexa Fluor 647)-CD206 (clone MR5D3), (PE)-PD-1 (clone J43) and (Pacific Blue)-Granzyme B (clone GB11). All antibodies were purchased from BD Biosciences or BioLegend (only Granzyme B). Stained cells were analyzed on a CytoFLEX S flow cytometry gating strategy flow cytometry gating strategy flow cytometer (BECHMAN COULTER). Data were analyzed using CytExpert software. Flow cytometry gating strategy were showed in **Figure S8**.

### Liver, kidney function and blood chemistry detection

Mice were sacrificed when tumors grew to 1500 mm^3^ and then blood was collected via mice eye. Blood was stored at 4 °C overnight and centrifugated at 1000 rpm to collect the serum. Livers were harvested and fixed in a 4% formalin in PBS solution, then embedded in paraffin, sectioned to 3-5 µm, floated on a water bath, picked up onto glass slides, and placed in slide racks for haematoxylin and eosin staining 42.

### CD8^+^ T cells isolation *in vivo*

Lymph nodes were crushed using a sterile syringe piston and screened with a 70 μm cell filter in PBS with 1 mM EDTA and 2% FBS at 4 °C. Tumors were finely minced and digested in RPMI 1640 with Mouse tumor tissue dissociation kit (Miltenyi, 130-096-730), then inverted in the tissue dissociator to run program as the protocol. Cell suspensions were screened with a 70 μm cell filter. Cells were centrifuged at 400 g for 10 min at 4 °C and re-suspended in PBS. T cells were isolated from tumor and lymph nodes by positive selection with magnetic Dynabeads CD8 (Invitrogen, 11447D), per the manufacturer's protocol.

### *In vivo* depletion

For cellular subsets depletion experiments, mice were injected intraperitoneally with 400 μg depletion antibody twice weekly beginning 1 day before therapy. The depletion antibody are as follows: anti-CD4 (clone GK1.5, BioXCell), anti-CD8α (clone 2.43, BioXCell), anti-NK1.1 (clone PK136, BioXCell) antibodies and isotype control antibody (clone LTF-2, BioXCell) 16. The depletion efficacy was confirmed by flow cytometry of PBMC on day 14 (**Figure S9**).

### Statistical analysis

Statistical analyses were performed using GraphPad Prism 8.0 software package. Difference between groups were analyzed as detailed in figure legends. Comparisons between groups were analyzed with one-way ANOVA. The significant differences were represented by P-values: n.s.* P*>0.05; * P <0.05; ** P <0.01; *** P <0.001; **** P < 0.0001.

## Results and Discussion

### Optimization of cdG/Mix delivery systems

cDNs activate the STING pathway, leading to the production of IFN-I and proinflammatory cytokines that play a critical role in antitumor immunity 43-45. RAW-Lucia ISG cells with an IFN regulatory factor (IRF)-inducible reporter were selected, in which IFN-I induction as an indicator to evaluate the tumor immunity ability of cdG-containing formulations.

**Figure 1:**
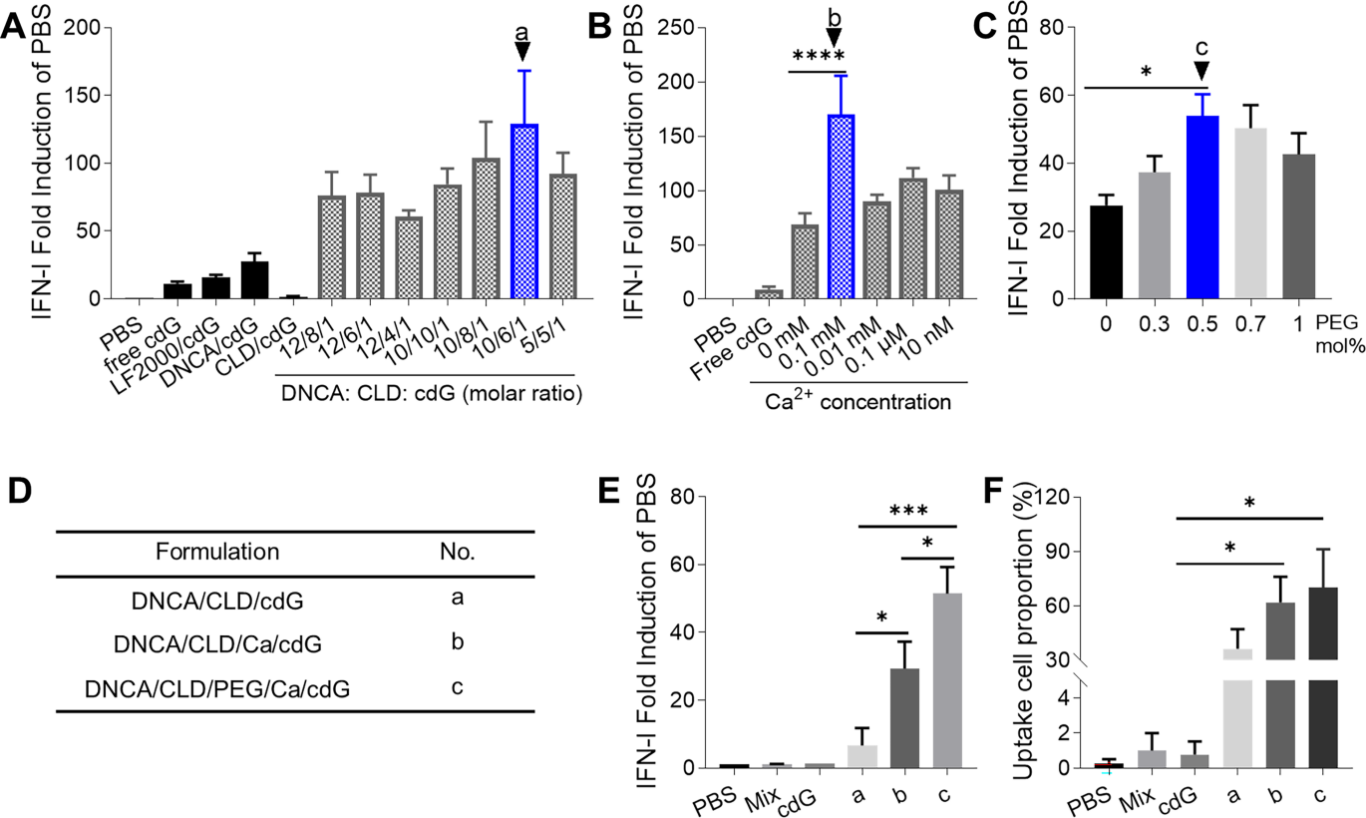
** The optimization of cdG delivery system. (A)** The optimization of molar ratio of DNCA and CLD (n=3). **(B)** The optimization of concentration of Ca^2+^ in cdG/Mix liposome (n≥4). **(C)** The optimization of PEG2000-DSPE mol% in cdG/Mix liposome (n≥3). **(D)** Description and No. of optimized formulations obtained from A-C. **(E)** IFN-I fold induction of variant cdG-containing formulations (n=2). **(F)** The uptake cell proportion of variant cdG-containing formulations (n≥3). cdG 1.4 µM in A and B, cdG 500 nM in C-F. A-F. One-way ANOVA, values are the mean ± SEM.

In the first round of cell level screening, a formulation of DNCA/CLD/cdG = 10/6/1 (molar ratio, formulation **a**) in PBS was found to be the best, which stimulated 10 fold IFN-I response compared with that of free cdG in RAW-Lucia ISG cells (**Figure 1A**, **2B**). Then with additional 0.1 mM Ca^2+^ in **a** (formulation **b**), IFN-I induction increased two-fold relative to that of **a** (**Figure 1B**). Addition of 0.5 mol% PEG2000-DSPE in **b** (formulation **c**) further increased 100% IFN-I induction compared with formulation **b** (**Figure 1C-E**). With fluorescently-labeled cdG (cdG-Dy547), formulation **c** showed the highest proportion of cellular uptake (**Figure 1F**).

### Characterization of cdG/Mix nanoparticles

**Figure 2:**
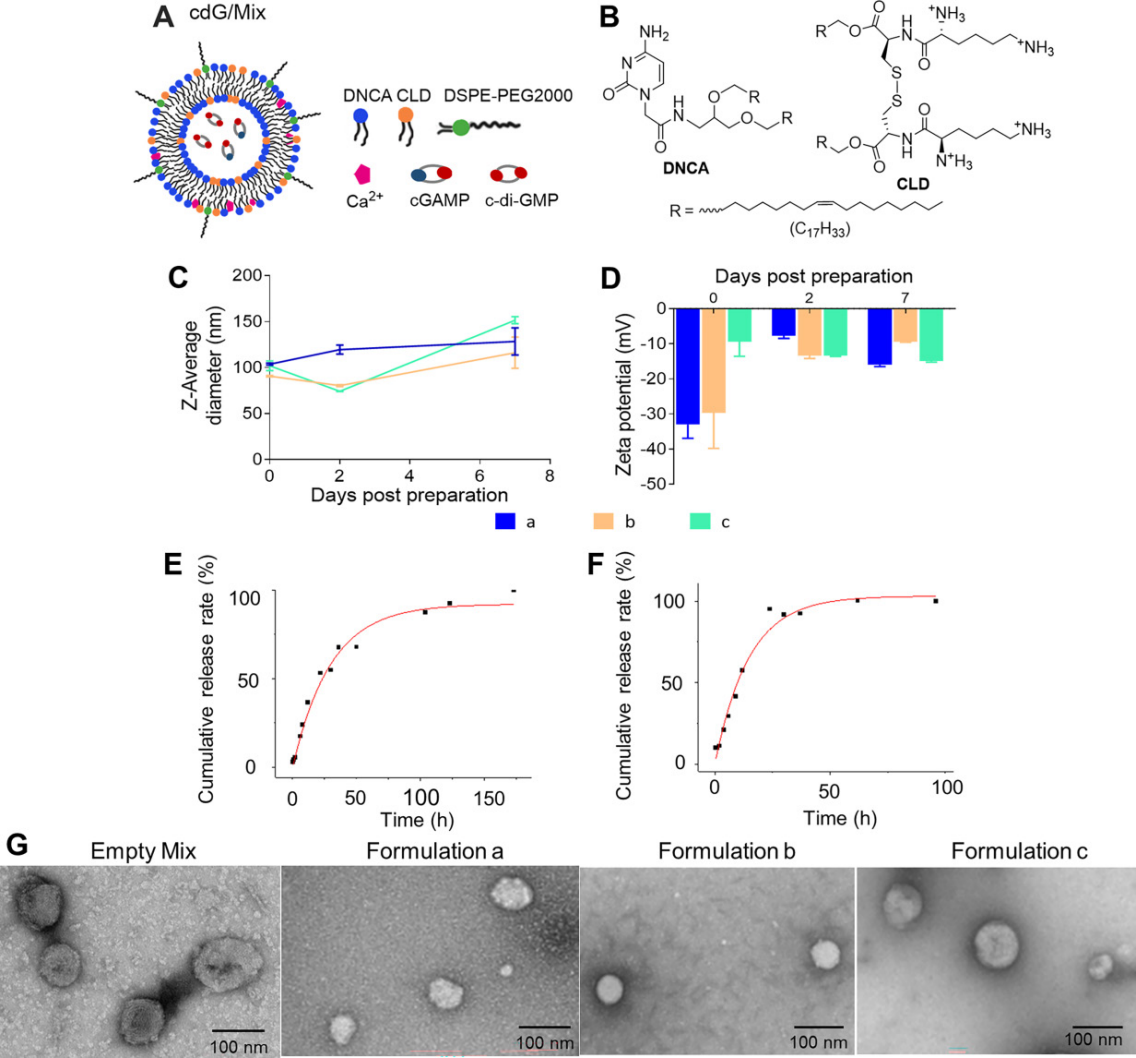
** The schematic diagram and characterization of nanoparticles. (A)** The schematic diagram of cdG/Mix (formulation **c**). **(B)** The chemical structures of the DNCA and CLD. The size **(C)** and Zeta potentials **(D)** of various nanoparticles measured by dynamic light scattering (DLS) for 7 days (n=3). Cumulative-release profile of cdG from formulation C incubated with PBS **(E)** and 10% FBS **(F)** at 37 °C (n = 3). **(G)** Representative transmission electron micrographs of indicated cdG-containing preparations. Formulation a: DNCA/CLD/cdG; Formulation b: DNCA/CLD/ Ca/cdG; Formulation c: DNCA/CLD/Ca/PEG/cdG.

Transmission electron micrographs (TEM) images reveal that indicated lipid can self-assemble as spherical vesicles with cdG and nanoparticle diameters were approximately 50-100 nm (**Figure 2G**). Consistent with previous observations, dynamic light scattering (DLS) data demonstrate that the size of three indicated formulations were homogenous (PDI < 0.3) at approximately 100 nm, although particle size increased gradually with the addition of composition ingredients (**Figure 2C-D, Table 1**). The zeta potential of formulations was all negative, however, formulation **c** was close to electrically neutral (-9.37 ± 7.23 mV) and exhibited higher loading capacity (5.7%) and encapsulation efficiency (94.5%, Table 1). The *in vitro* stability of these three nanoparticles were evaluated by monitoring the particle size and Zeta potential for 7 days at 4 °C. The results showed that the particle size of the three formulations did not change much, but the Zeta potential of formulations **a** and **b** changed significantly, and only formulation **c** remained stable (**Figure 2C-D**). The release behavior of cdG from various formulation were assessed in PBS (pH 7.4) and 10% fetal bovine serum (FBS) at 37 °C (**Figure 2, S1**). The formulation **c** showed nonlinear release of cdG both in PBS and 10% FBS. Formulation **c** achieved slow release of cdG; 50% cdG was released within 25 h and 98% was released within 7 days in PBS (**Figure 2E**); 50% cdG was released within 12 h and 100% was released within 4 days in 10% FBS, showing a faster release profile compared to that in PBS (**Figure 2F**). However, the formulation **a** and **b** both exhibited faster release rate, 50% cdG was released within 5 h and 100% was released within 40 h (**Figure S1**). Given that formulation **c** (hereafter referred to as cdG/Mix) possessed higher loading and encapsulation efficiency, slower release rate of drug and better *in vitro* stability, cdG/Mix (**Figure 2A**) was selected as the optimal formulation for the cDNs delivery and was used for all subsequent studies.

### cdG/Mix triggers tumor cell apoptosis and immunogenic cell death in both human and mouse tumor cells

**Figure 3:**
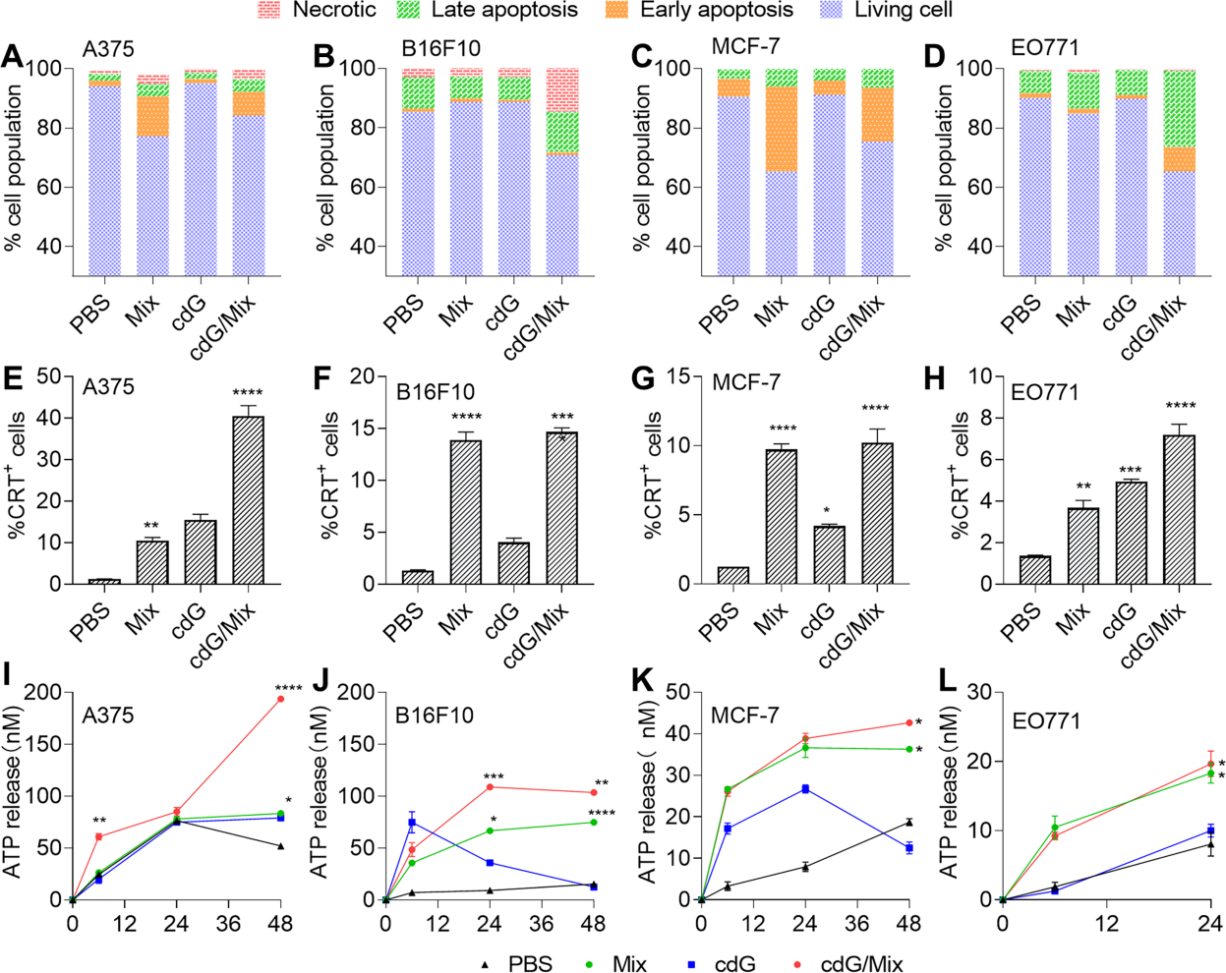
Quantification of the cell apoptosis induced by indicated formulations after 24 h in A375 **(A)**, B16F10 **(B)**, MCF-7 **(C)** and EO771 **(D)** (n=3). Flow cytometric analysis of cell surface calreticulin (CRT) on A375 **(E)**, B16F10 **(F)**, MCF-7 **(G)** and EO771 **(H)** cells in response to indicated formulations after 24 hours (n=3, one-way ANOVA). ATP release in A375 **(I)**, B16F10 **(J)**, MCF-7 **(K)** and EO771 **(L)** following treatment with various formulations for 6, 24 and 48 hours (n=3, two-way ANOVA). cdG 500 nM in A375 and B16F10 cells related experiments; cdG 800 nM in MCF-7 cells related experiments; cdG 1.4 µM in E0771 cells related experiments. All data shown are mean ± SEM. Difference (*) denote the significance levels of indicated group versus PBS group.

Annexin V/PI staining revealed that both Mix and cdG/Mix treatment induced higher levels of apoptosis in human and mouse melanoma (A375 and B16F10) and breast cancer (MCF-7 and EO771) cells (**Figure 3A-D**) relative to PBS. Nevertheless, Mix induced limited apoptosis in B16F10 (**Figure 3B**).

ICD promoted antigen cross-presentation and provided an inflammatory context for generating antigen-specific T cell responses 21. The hallmarks of ICD, including cell-surface calreticulin (CRT) and adenosine triphosphate (ATP) release, were significantly elevated on A375, B16F10, MCF7 and EO771 cells (**Figure 3E-L**), suggesting that both Mix and cdG/Mix triggered ICD in indicated cancer cell lines. Notably, neither Mix nor cdG/Mix elicited any signature of apoptosis in normal cells, including HFL-1 (human lung epithelial fibroblasts) cells and RAW264.7 cells (**Figure S2**).

### Enhanced immunostimulatory activity of cdG/Mix etc. *in vitro* and *in vivo*

**Figure 4:**
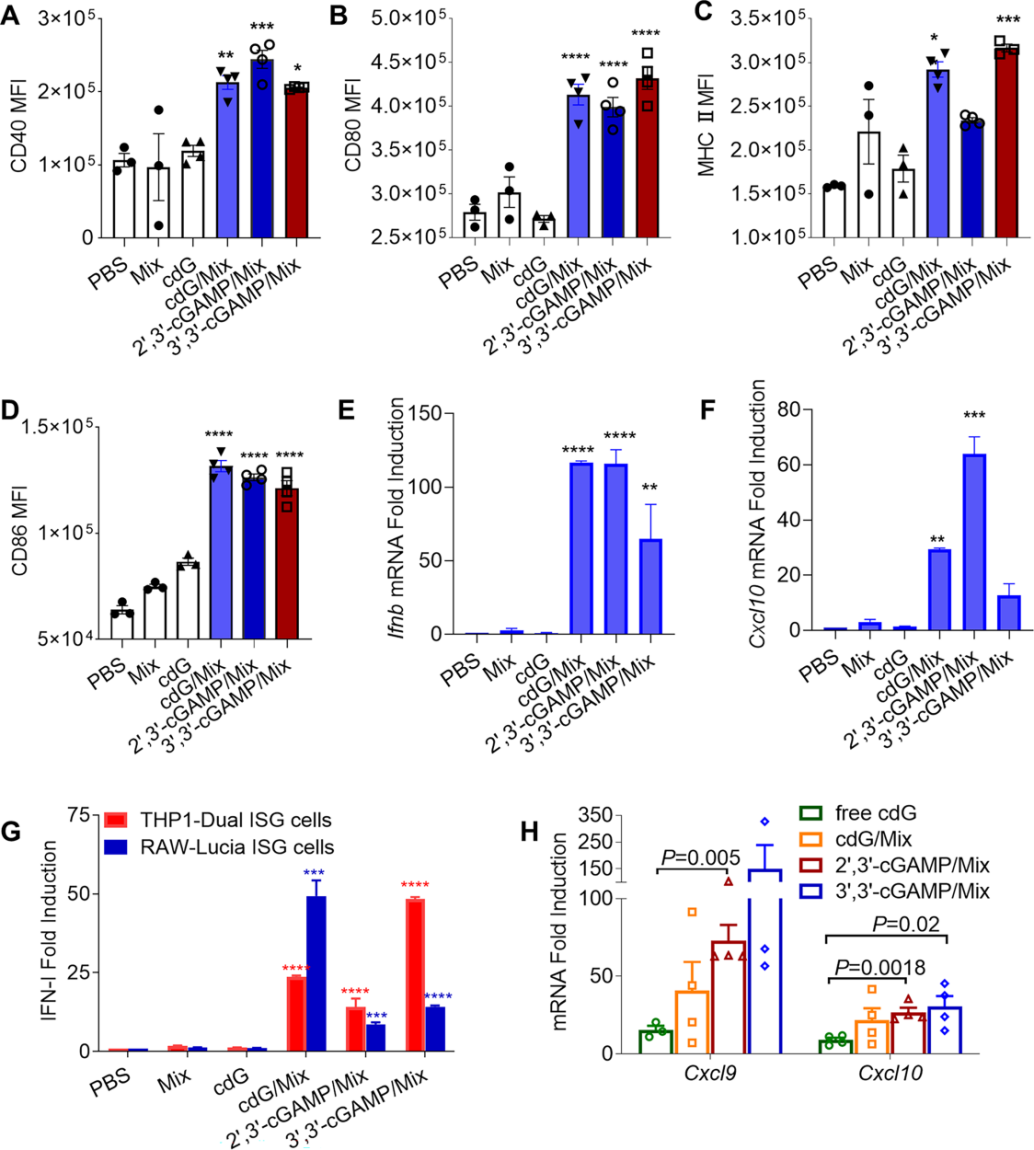
Flow cytometric quantification of CD40 **(A)**, CD80 **(B)**, MHC-II **(C)** CD86 **(D)** expression in BMDCs treated with indicated formulations treated for 24 h (cdG etc.: 500 nM, n=3-4). RT-qPCR analysis of *Ifnb* **(E)** and *Cxcl10* **(F)** mRNA expression in Raw264.7 cells after 4 h incubation with encapsulated 500 nM cdG etc. (n=3). **(G)** IFN-I fold induction of encapsulated 500 nM cdG etc. in RAW-Lucia ISG cells and THP1-Dual ISG cells after 18 h incubation (n=3-6). **(H)** RT-qPCR analysis of *Cxcl9* and *Cxcl10* mRNA expression in B16F10 tumors at 8 h after intratumoral administration of PBS, cdG (5 µg), 2',3'-cGAMP(5 µg)/Mix, 3',3'-cGAMP (5 µg)/Mix and cdG (5 µg)/Mix (n=3-4). A-H, one-way ANOVA, data shown as mean±SEM. Difference (*) denote the significance levels of indicated group versus cdG group.

DC maturation is critical for antigen presentation and the initiation of subsequent T cell immune responses 46. To test the ability of cdG/Mix etc. to promote the maturation of DCs, naïve BMDCs was treated for 24 h with indicated formulations. Notably, cdG/Mix 3',3'-cGAMP/Mix and 2',3'-cGAMP/Mix all significantly increased the expression of CD40, MHCII, CD80 and CD86 in BMDCs around 2-fold compared with free cdG (**Figure 4A-D**).

In addition, the three natural cDNs (cdG, 3',3'-cGAMP and 2',3'-cGAMP) encapsulated by Mix elicited substantial IFN-I release in RAW-Lucia ISG and THP1-Dual ISG cells relative to PBS and free cdG (**Figure 4G**). Consistent with this, the cdG/Mix etc. led to significantly upregulated *Ifnb* and* Cxcl10* mRNA levels in RAW cells relative to PBS and free cdG (**Figure 4E-F**). Furthermore, cdG/Mix, 3',3'-cGAMP/Mix and 2',3'-cGAMP/Mix all significantly up-regulated *Cxcl9* and* Cxcl10* mRNA level in the TME of B16F10-burdened mice by 3 to 15-fold compared to free cdG, which were critical chemokines of antitumor T-cell activation and recruitment (**Figure 4H**).

### Enhanced anti-tumor efficacy of cdG/Mix etc. in the B16F10 melanoma model

**Figure 5:**
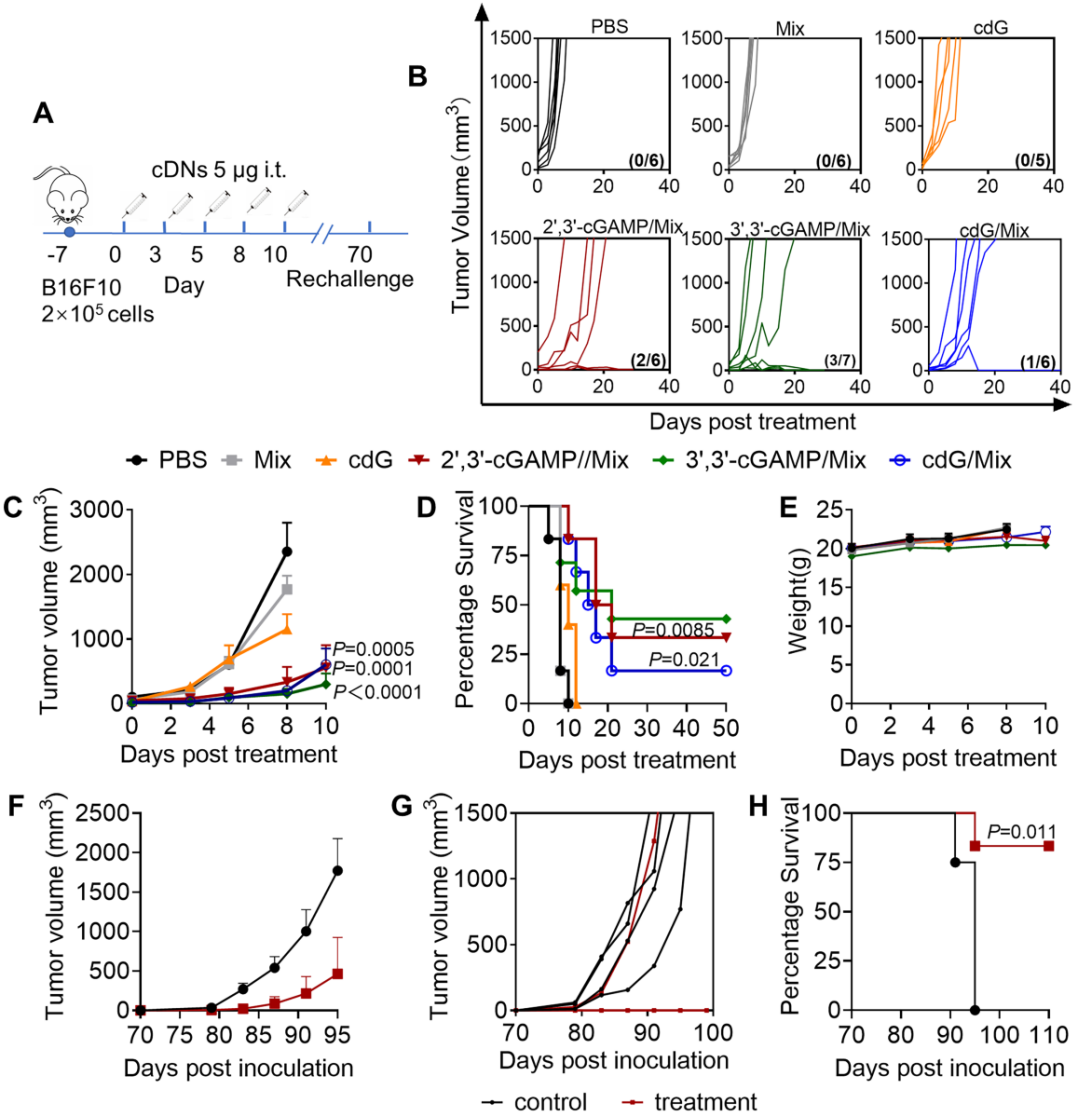
** Enhanced immunotherapeutic efficacy of cdG/Mix etc. (A)** Intratumoral administration and tumour rechallenge scheme for mice with B16F10 tumor. Mice with subcutaneous tumours were treated with PBS, empty Mix, cdG, 2',3'-cGAMP/Mix, 3',3'-cGAMP/Mix and cdG/Mix intratumorally five times in 10-day treatment period. Mice that cleared their tumors by cDNs treatment were injected subcutaneously in the flank on day 70 with 10^5^ B16-F10 cells and monitored for survival. **(B)** Spider plots of individual tumor growth curves, with the complete response proportion denoted. **(C)** Mean tumour volume of mice treated with indicated formulations (n = 5-7 mice/group; *P* = 0.0005, *P* = 0.0001 and *P* < 0.0001 denote the significance levels of 2',3'-cGAMP/Mix, cdG/Mix and 3',3'-cGAMP/Mix group, respectively, versus cdG group; two-way ANOVA). **(D)** Kaplan-Meier survival curves of mice treated with the indicated formulation (n=5-7; *P* = 0.021 and *P* = 0.0085 denote the significance of 2',3'-cGAMP/Mi and cdG/Mix from cdG, Mantel-Cox test). **(E)** Mean body weight of mice treated with indicated formulations. Mean **(F)** and individual **(G)** tumor volume of treatment naïve mice and mice showing complete responses to cdG/Mix etc., which were rechallenged with B16F10 cells 70 d after the treatment.** (H)** Kaplan-Meier survival curves of mice rechallenged with B16F10 cells (Mantel-Cox test). All data shown are mean ± SEM.

Mice were treated with cdG (5 μg)/Mix etc. (i.t.) when tumors were palpable and then treated according to scheme with a total of five injections (**Figure 5A**). Once the last injection was complete, mice were monitored continuously until they reached the study endpoint (1500 mm^3^). Administration with the cdG/Mix etc. effectively inhibited tumor growth relative to PBS and free cdG (**Figure 5B-C**). 2',3'-cGAMP/Mix, 3',3'-cGAMP/Mix and cdG/Mix showed equal efficacy on tumor progression inhibition and significantly increased the long-term survival rate of B16F10 murine models to 33.3% (2/6), 42.9% (3/7) and 16.7% (1/6), respectively, and no tumor burden was observed until 50 days post the treatment (**Figure 5D**). These survival mice was rechallenged with 10^5^ B16F10 tumor cells and tumor volume was monitored. Five of six (83%) rechallenged mice showed no tumor growth through at least 110 days without any additional treatment compared to age-matched control mice (**Figure 5F-G**). During the treatment period, the mice gained moderate weight and the blood biochemical indexes of liver and kidney functions were within the normal extent (**Figure 5E, S3**).

### cdG/Mix enhance the anti-tumor immunology activity at lower doses by alternative route of administration

**Figure 6:**
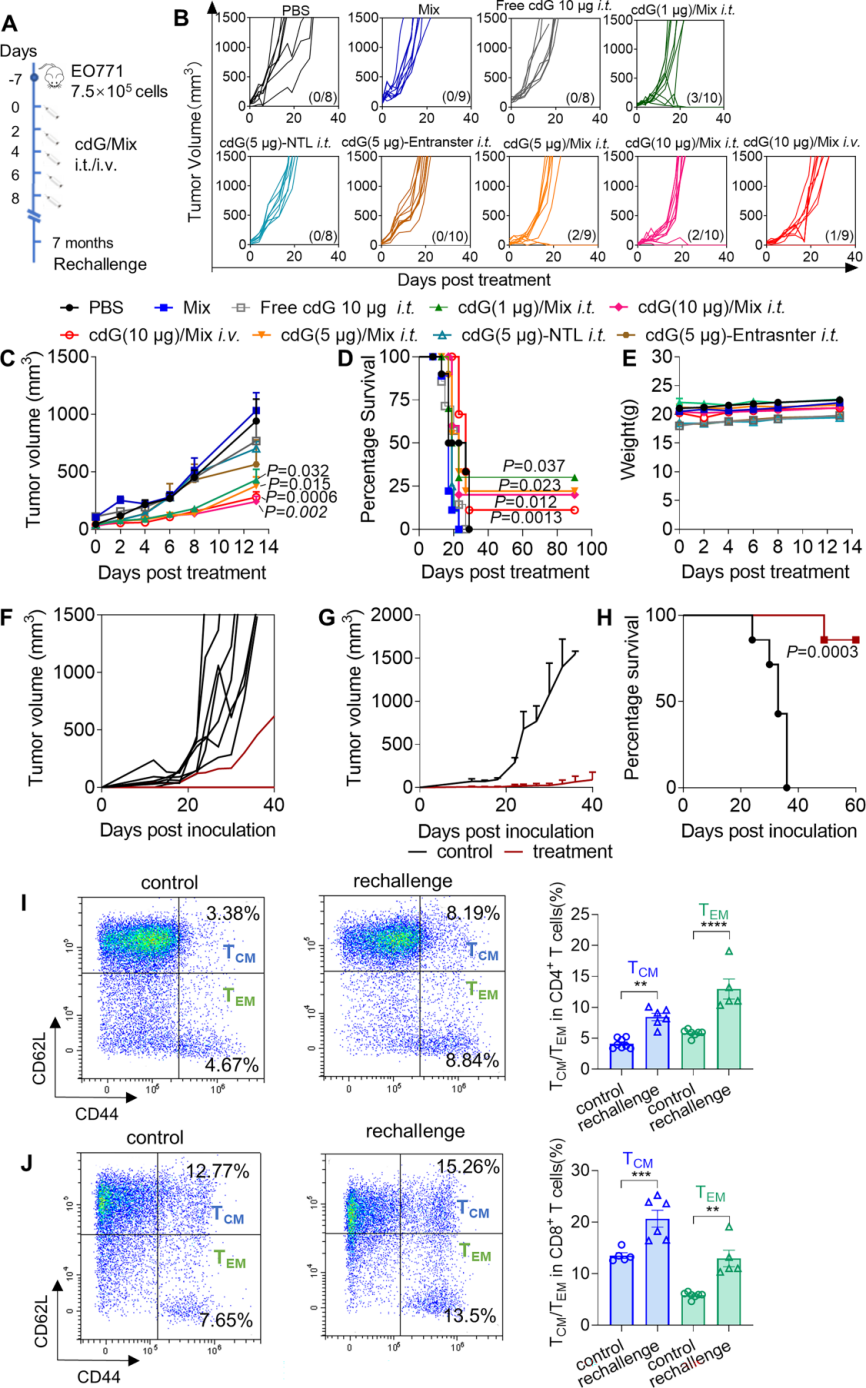
** cdG/Mix enhance the immunotherapeutic efficacy at lower dosage with various administration routes. (A)** cdG/Mix administration and tumor re-challenge scheme for mice with EO771 tumor. Mice with 100 mm^3^ subcutaneous tumors were administered PBS, empty Mix, cdG (1, 5 and 10 µg)/Mix (i.t.) and cdG (10 µg)/Mix (i.v.) five times, 2 days apart. cdG (5 µg)-NTL and cdG (5 µg)-Entranster are control preparations. Mice that cleared their tumors by treatment were injected subcutaneously in the flank 7 months later and monitored for survival. **(B)** Spider plots of individual tumor growth curves, with the complete response proportion denoted. **(C)** Mean tumor volume of mice treated with various formulations (n=8-10 mice/group; the significance of cdG (1, 5 and 10 µg)/Mix intratumorally and cdG (10 µg)/Mix intravenously versus PBS is *P=*0.032, *P=*0.015, *P=*0.002 and *P=*0.0006; two-way ANOVA). **(D)** Kaplan-Meier survival curves of mice treated with the indicated formulation using a 1500 mm^3^ tumor volume as the endpoint criteria (*P* = 0.037, *P* = 0.023, *P* = 0.012 and *P* = 0.0013 denote the significance levels of cdG (1, 5 and 10 µg)/Mix (i.t.) and cdG (10 µg)/Mix (i.v.), respectively, versus PBS group; Mantel-Cox test). **(E)** Mean body weight of mice following treatment with various dosage of cdG/Mix. Mean **(F)** and individual **(G)** tumor volume of treatment naïve mice and mice showing complete responses to cdG/Mix in rechallenge experiment (n = 7 or 8). **(H)** Kaplan-Meier survival curves of mice rechallenged with EO771 cells (n=7 or 8; Mantel-Cox test). Representative scatter plots and quantification of the percentage of CD4^+^ **(I)** and CD8^+^** (J)** Tcm and Tem cells in blood of the rechallenged mice. All data shown are mean ± SEM.

The EO771 model, a kind of TNBC model that was the most aggressive form of breast cancer and lacked effective targeted therapy 47, was chose to evaluate the multiple anti-tumor activity of cdG/Mix and explore a lower dosage with optimal curative effect in tumor-burdened mice. cdG (1 μg)/Mix (i.t.) administration significantly inhibited tumor growth and showed equal anti-tumor efficacy to 5 and 10 μg cdG/Mix, achieving an almost 10-fold reduction of effective dose (**Figure 6A-C**). Importantly, treatment with cdG (1, 5 and 10 μg)/Mix (i.t.) significantly increased the survival rate of EO771 tumor model to 30%, 22% and 20%, respectively, and no tumor burden was observed until 90 days post treatment (**Figure 6D**). cdG (5 μg)/Mix (i.t.) showed significant reduction in tumor volume compared to PBS (*P* = 0.015), however, control preparations, cdG-NTL and cdG-Entranster, showed limited benefits in tumor suppression and survival of mice, suggesting the advantages of Mix delivery system (**Figure 6C-D**) 39. 3',3'-cGAMP delivered with Ace-DEX MPs (polymer) can inhibit tumor growth at the lowest dosage (0.1 μg i.t., seven injections) reported so far with an inferior long-term survival rate (10%) 16. However, 3',3'-cGAMP (10 μg) showed stronger anti-tumor efficacy over other pathogen associated molecular patterns when delivered with Ace-DEX MPs 16. cdG (1 μg)/Mix (i.t.) maintained a higher long-term survival rate at a lower dose and injection frequency, achieving a balance between dose and anti-tumor efficacy. cdG (10 μg)/Mix (i.v.) significantly inhibited tumor growth, showing equal anti-tumor efficacy to intratumoral administration (**Figure 6B-D**) and the long term-survival rate increased to 11% (**Figure 6D**). These results demonstrate that cdG/Mix (i.v.) could serve as an alternative route of administration to patients with non-accessible tumors, providing promising and durable anti-tumor activity and moderate survival benefit. During the treatment period, the mice gained moderate weight and the blood biochemical indexes of liver and kidney functions were within the normal extent (**Figure 6E, S4**).

Next, these survival mice were rechallenged with 3.5×10^5^ EO771 cells and monitored tumor volume. Seven of eight (87.5%) rechallenged mice showed no tumor growth through at least 60 days post rechallenge without any additional treatment and the remaining one mouse showed slower tumor growth than controls (**Figure 6F-H**). Fourteen days post rechallenge, the blood of living rechallenged mice were collected and analyzed to estimate the percentages of CD8^+^ or CD4^+^ central memory T (Tcm, CD44^high^CD62L^high^) cells and effector memory T (Tem, CD44^high^CD62L^low^) cells. cdG/Mix significantly improved the proportion of Tem and Tcm in CD8^+^ and CD4^+^ T cells among rechallenged mice (**Figure 6I-J**), which revealed that cdG/Mix induced the immune memory effects in mice to against tumor recurrence.

### cdG/Mix boosts NK and CD8^+^ T cell activation in tumor and systemic immune organization

**Figure 7:**
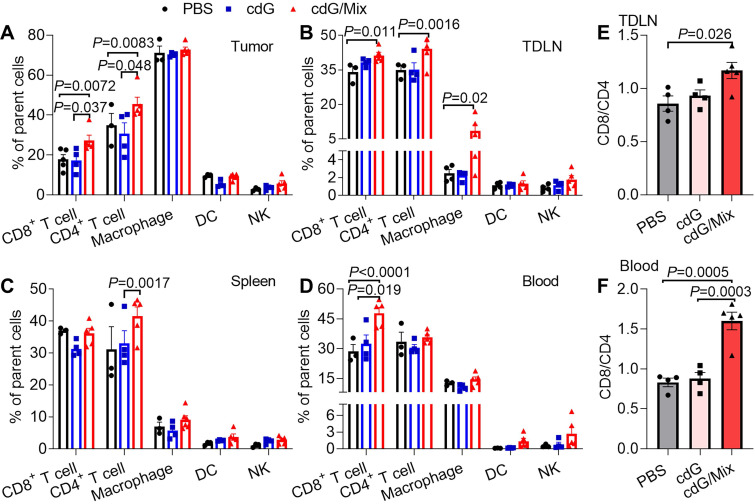
Flow cytometry analysis of the proportion of immune cell subsets in the TME (**A**), TDLN (**B**), spleen (**C**) and blood (**D**) of EO771-tumor-bearing mice 48 h after single dose of intratumoral treatment (n = 3-5; one-way ANOVA). Ratio of CD8^+^ to CD4^+^ T cells in the TDLN (**E**) and blood (**F**) (n = 3-5; one-way ANOVA). cdG (5 µg) and cdG (5 µg)/Mix. All data shown are mean ± SEM.

The impact of cdG/Mix on the immunocellular composition of tumor and systemic immune organization in EO771 tumor-bearing mice was explored either. Mice were inoculated with EO771 tumor cells and treated with a single dose of PBS, cdG (5 μg) or cdG (5 μg)/Mix. Mice were sacrificed 48 h after administration, and then tumors, tumor-draining lymph node (TDLN), spleen and peripheral blood were harvested for subsequent flow cytometry. A single intratumoral injection of cdG/Mix substantially increased the fraction of infiltrating CD8^+^ and CD4^+^ T cells in TME and TDLN relative to free cdG and PBS (**Figure 7A-B**). cdG/Mix also increased the proportion of CD8^+^ T cells in blood and CD4^+^ T cells in the spleen (**Figure 7C-D**). Furthermore, the CD8^+^/CD4^+^ ratio of TDLN and blood were significantly increased in the cdG/Mix group, a commonly reported independent favorable prognostic factor (**Figure 7E-F, S5**) 48-50. No significant fraction changes occur in the other leukocyte populations (**Figure 7A-D**).

**Figure 8:**
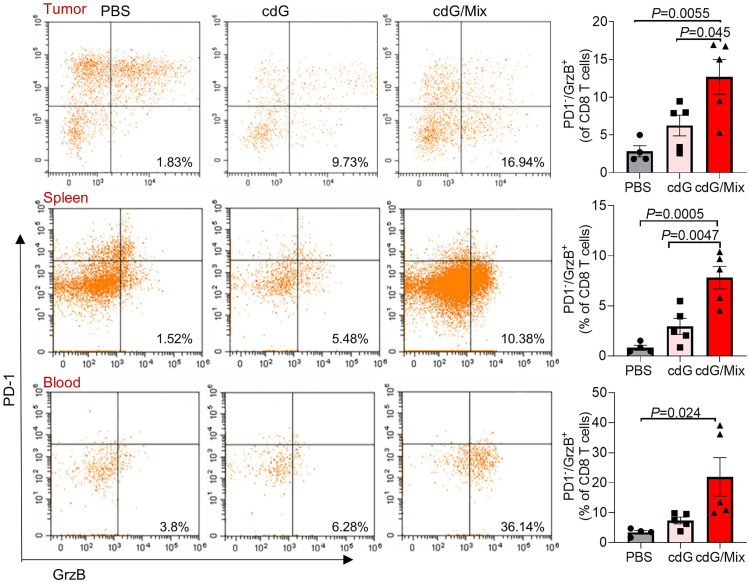
Representative scatter plots and quantification of the proportion of Granzyme B^+^ PD-1^-^ CD8^+^ T cells in the TME, spleen and blood of EO771-tumor-bearing mice 48 h after single dose of indicated formulation by intratumoral administration. All panels are from one experiment (n = 4-5; one-way ANOVA). cdG (5 µg) and cdG (5 µg)/Mix. All data shown are mean ± SEM.

**Figure 9:**
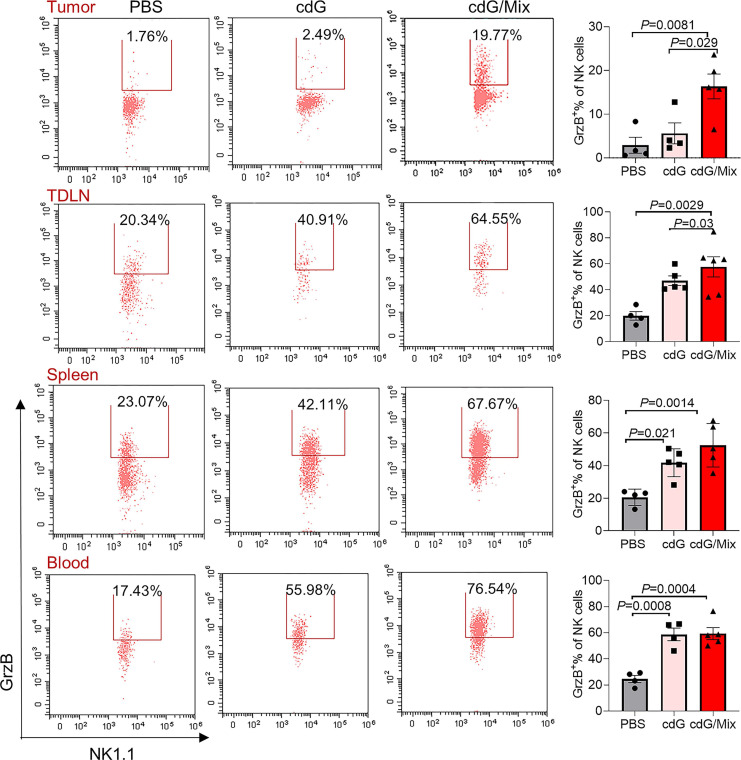
Representative scatter plots and quantification of the proportion of Granzyme B^+^ NK cells in the TME, TDLN, spleen and blood of EO771-tumor-bearing mice 48 h after single dose of indicated formulation by intratumoral administration. All panels are from one experiment (n = 3-5; one-way ANOVA). cdG (5 µg) and cdG (5 µg)/Mix. All data shown are mean ± SEM.

Additionally, activated cytotoxic CD8^+^ T cells (defined as granzyme B^+^ PD-1^-^) significantly accumulated within the TME, spleen and blood of mice treated by cdG/Mix, which is a positive indicator of survival in patients with resected disease and response to immunotherapy in advanced cancer cases (**Figure 8**) 51. The granzyme B^+^ PD-1^-^ CD8^+^ T cells also increased within TDLN although the increase was not statistically significant (**Figure S6**). Meanwhile, the percentage of granzyme B^+^ NK cells within tumor and immune systems also significantly increased, suggesting that cdG/Mix induced activation of cytotoxic NK cells both within tumors and systemically relative to PBS and cdG, although the percentage of NK cells was not significantly increased (**Figure 7A-D, 9**).

cdG/Mix treatment mainly induced activation and recruitment of CD8^+^ T and NK cells within tumors and to some extent systemically, consistent with previous observations that cdG/Mix caused the immediate climbing of *Cxcl9* and *Cxcl10* chemokines responsible for driving the trafficking of CD8^+^ T cells and NK cells 52 mRNA expression in tumors (**Figure 4H**).

### CD8^+^ T cells were critical to anti-tumor immune response generated by cdG/Mix

**Figure 10:**
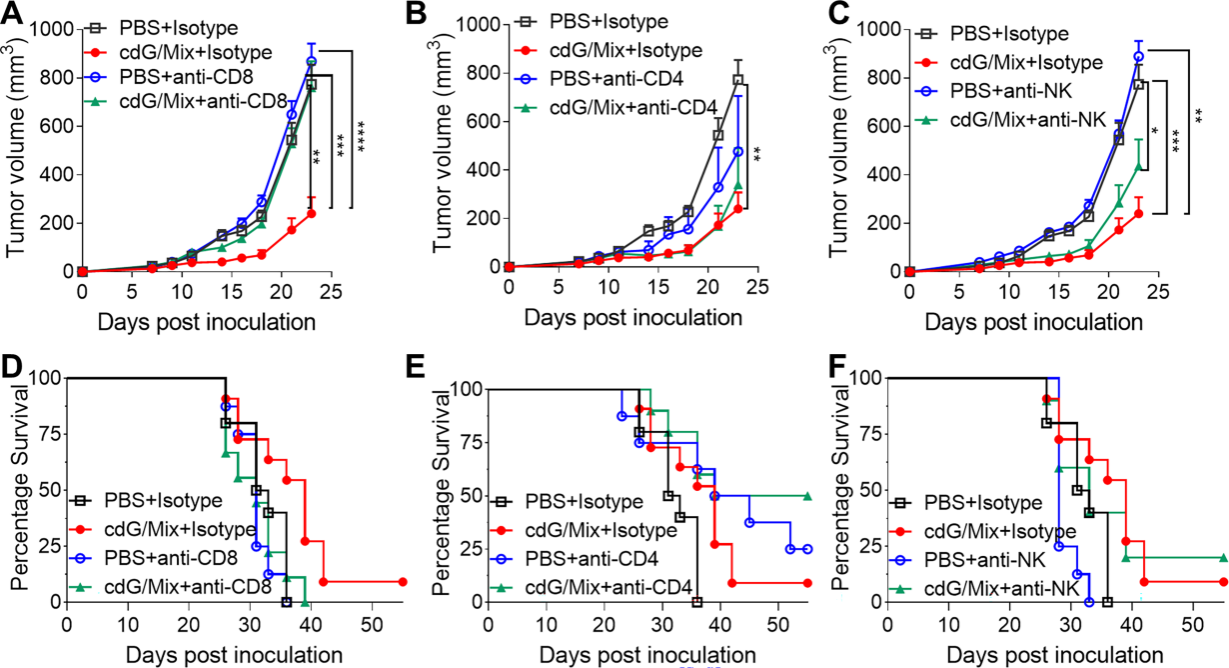
** CD8^+^ T cells were critical to anti-tumor immune response generated by cdG/Mix. (A-C)** Mean tumor volume of mice in indicated depletion groups. **(D-F)** Kaplan-Meier survival curves of mice in indicated depletion groups. C57BL/6 mice (n = 10 animals/group for isotype control, n =8 animals/group for PBS + antibody depletion, n = 10 animals/group for cdG/Mix + antibody depletion) were inoculated with 7.5 × 10^5^ EO771 cells s.c. in the flank. Depletion antibodies (400 µg) were administered i.p. beginning one day prior to initiation of cdG (5 µg)/Mix treatment (i.t., five times, 2 days apart). The isotype control groups are repeated in graph. A-C. Two-way ANOVA, data shown as mean ± SEM, * *P* <0.05; ** *P* <0.01; *** *P* <0.001; **** *P* < 0.0001.

Antibody depletion studies were performed to define the key cells for anti-tumor effect induced by cdG/Mix in the E0771 mouse. *In vivo* depletion experiments showed that tumor volume in mice treated with cdG/Mix + anti-CD8 depletion antibody was significantly bigger than that in mice treated with cdG/Mix + Isotype and was almost identical to that of PBS + Isotype group (**Figure 10A**). In addition, anti-CD8 depletion also led to significant reductions in overall survival rates and median survival compared to cdG/Mix + Isotype group (**Figure 10D**). The above results demonstrated that CD8^+^ T cells were critical to tumor rejection in cdG/Mix treatment. Tumor volume in mice treated with cdG/Mix + anti-NK depletion antibody was bigger than that in mice treated with cdG/Mix + Isotype and was significantly smaller to that of PBS + Isotype group, suggesting that NK cells contribute partially to tumor inhibition in cdG/Mix treatment (**Figure 10C, E**). In contrast, mice treated with cdG/Mix + anti-CD4 depletion antibody showed similar tumor volume to that in mice treated with cdG/Mix + Isotype and significantly increased the longterm-survival rate to 50% (**Figure 10B, E**), possibly due to the depletion of CD4^+^ T regulatory cells.

### cdG/Mix reverses T cell exhaustion in tumor and systemic immune organization

**Figure 11:**
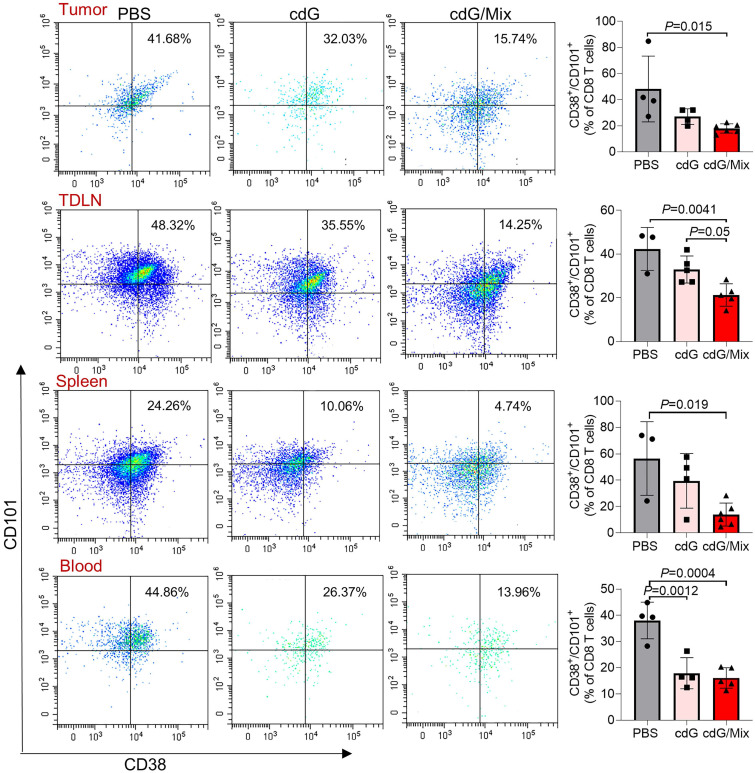
Representative scatter plots and quantification of the proportion of exhausted T cells (defined as CD101^+^ CD38^+^ CD8^+^) in the TME, TDLN, spleen and blood of EO771-tumor-bearing mice 48 h after single intratumoral injection of indicated formulation. Mice were inoculated with the EO771 tumor cells and treated with single dose of PBS, cdG (5 µg) and cdG (5 µg)/Mix intratumorally. Mice were then sacrificed 48 h after administration and tumors, TDLN, spleen and peripheral blood were harvested for subsequent flow cytometry. All panels are from one experiment (n = 3-5; one-way ANOVA). All data shown are mean ± SEM.

Tex is common in tumors, along with a state of impaired effector functions, high expression of inhibitory receptors, transcriptional reprogramming, and defective immune memory, all of which prevent optimal control of tumors 36. Here, the potential of cdG/Mix to reverse Tex within tumor and the systemic immune system in EO771 tumor-bearing mice was explored by detecting the expression of CD101 and CD38, which were recently identified as evidence of permanent T cell dysfunction 41,53. cdG/Mix significantly decreased the CD101^+^ CD38^+^ CD8^+^ T cell proportions among tumor and systemic immune organization relative to PBS and free cdG, suggesting cdG/Mix can reverse Tex in TME and even, to some extent, systemically (**Figure 11**).

**Figure 12:**
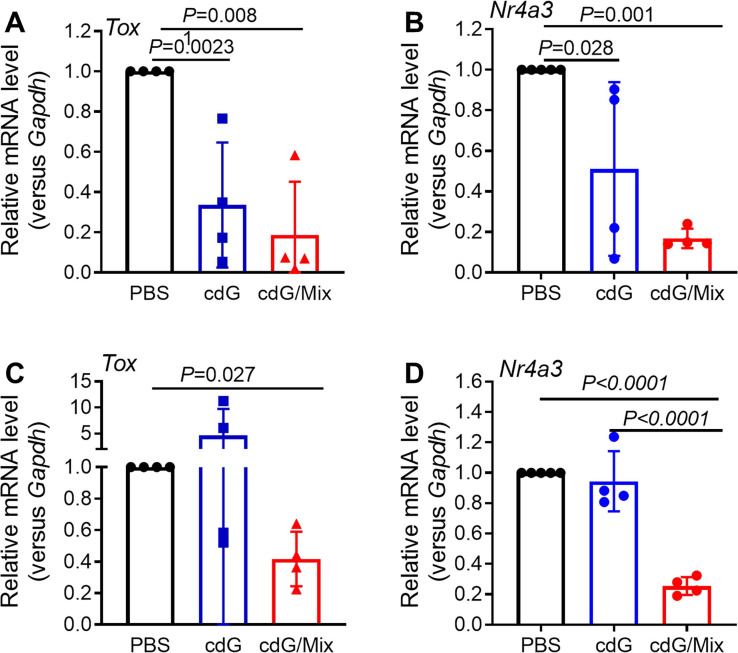
RT-qPCR analysis of *Tox* **(A)** and *Nr4a3* **(B)** mRNA expression in CD8^+^ T cells isolated from tumor tissues of EO771-burdened mice. RT-qPCR analysis *Tox* **(C)** and *Nr4a3* **(D)** mRNA expression in CD8^+^ T cells isolated from TDLN of EO771-burdened tumor. Mice were inoculated with the EO771 tumor cells and treated with PBS, cdG (5 µg) and cdG (5 µg)/Mix intratumorally every other day for 5 times. Mice were sacrificed 48 h after last injection. Tumors and TDLN were harvested for subsequent qPCR analysis. A-D, n=4, one-way ANOVA, data shown as mean ± SEM.

TOX and NR4A were recently reported to be key transcription factors imposing CD8^+^ T cell exhaustion 54,55. *Tox* and* Nr4a* mRNA expression in the CD8^+^ T cells isolated from tumor and TDLN of EO771-burdened mice was detected at 48 h after treatment with cdG (5 μg)/Mix. cdG/Mix significantly inhibited the mRNA expression of *Tox* and* Nr4a* to ~20% - 40% both in tumor and TDLN relative to PBS, suggesting that cdG/Mix could interfere with the expression of *Tox* and *Nr4a* mRNA to reverse the Tex (**Figure 12 A-D**). Confocal results of RAW264.7 cells revealed that cdG-Dy547/Mix at 2 h showed co-localization with the nucleus in many cells, suggesting the possibility of cdG/Mix modulating *Tox* and *Nr4a* mRNA directly or indirectly in the nucleus (**Figure S7**).

## Conclusion

Mix, a novel delivery system, enhances the cytosolic delivery of cDNs, possessing an excellent anti-tumor immunology effect in two murine tumor models by inducing ICD of tumor cells. cDNs/Mix (i.t.) can significantly inhibit tumor growth, prolong survival of tumor-burden mice, induce the immune memory effects and lead to 83% and 87.5% of mice obtain the resistance to B16F10 and EO771 tumor, respectively. Compared with other reported delivery system, cDNs/Mix maintain a higher long-term survival rate at a lower dose, achieving a balance between dose and anti-tumor efficacy. In addition, cdG (10 μg)/Mix (i.v.) also significantly inhibited tumor growth with 11% long-term survival, offering a treatment opportunity to patients with non-accessible cancer. Furthermore, the cdG/Mix reversed the Tex systemically by interfering with the expression of* Tox* and* Nr4a*, leading to the potent recruitment and activation of NK and CD8^+^ T cells. In conclusion, cDNs/Mix could be a promising alternative strategy for cancer immunotherapy. Exploration of mechanism of cdG modulating Tex may shed new light on the development of immunotherapies.

## Supplementary Material

Supplementary figures and tables.

## Figures and Tables

**Table 1:** The characteristics of various formulation

No.	Formulation	Z-Average (d, nm)	Zeta Potential (mV)	PDI	LC%	EE%
**a**	DNCA/CLD/cdG	96.5 ± 0.27	-33.0 ± 6.78	0.245	4.3	71.3
**b**	DNCA/CLD/Ca/cdG	117 ± 0.25	-29.6 ± 17.6	0.251	4.8	79.2
**c**	DNCA/CLD/Ca/PEG/cdG	141 ± 0.28	-9.37 ± 7.23	0.266	5.7	94.5

LC, loading capacity; EE, encapsulation efficiency.

## Abbreviations

cDNs: cyclic dinucleotides
STING: stimulator of interferon genes
ICD: immunogenic cell death
Tex: T cell exhaustion
2ʹ,3ʹ-cGAMP: 2ʹ,3ʹ-cyclic guanosine monophosphate-adenosine monophosphate
cdG: cyclic diguanylate
IFN-Ⅰ: type I interferon
DNCA: neutral cytidinyl lipid
CLD: cationic lipid
TME: tumor environment
IRF: IFN regulatory factor
DLS: dynamic light scattering
PDI: polydispersity Index
FBS: fetal bovine serum
CRT: calreticulin
ATP: adenosine triphosphate
TDLN: tumor-draining lymph node

## References

[1] Wang Y, Luo J, Alu A, Han X, Wei Y, Wei X (2020). cGAS-STING pathway in cancer biotherapy. Mol Cancer.

[2] Meric-Bernstam F, Sweis RF, Hodi FS, Messersmith WA, Andtbacka RHI, Ingham M (2022). Phase I dose-escalation trial of MIW815 (ADU-S100), an intratumoral STING agonist, in patients with advanced/metastatic solid tumors or lymphomas. Clin Cancer Res.

[3] Pan B, Perera SA, Piesvaux JA, Presland JP, Schroeder GK, Cumming JN (2020). An orally available non-nucleotide STING agonist with antitumor activity. Science.

[4] Li T, Cheng H, Yuan H, Xu Q, Shu C, Zhang Y (2016). Antitumor Activity of cGAMP via stimulation of cGAS-cGAMP-STING-IRF3 mediated innate immune response. Sci Rep.

[5] Gulen MF, Koch U, Haag SM, Schuler F, Apetoh L, Villunger A (2017). Signalling strength determines proapoptotic functions of STING. Nat Commun.

[6] Sivick KE, Desbien AL, Glickman LH, Reiner GL, Corrales L, Surh NH (2018). Magnitude of therapeutic STING activation determines CD8^+^ T cell-mediated anti-tumor immunity. Cell Rep.

[7] Koshy ST, Cheung AS, Gu L, Graveline AR, Mooney DJ (2017). Liposomal delivery enhances immune activation by STING agonists for cancer immunotherapy. Adv Biosyst.

[8] An M, Yu C, Xi J, Reyes J, Mao G, Wei W (2018). Induction of necrotic cell death and activation of STING in the tumor microenvironment via cationic silica nanoparticles leading to enhanced antitumor immunity. Nanoscale.

[9] Junkins RD, Gallovic MD, Johnson BM, Collier MA, Watkins-Schulz R, Cheng N (2018). A robust microparticle platform for a STING-targeted adjuvant that enhances both humoral and cellular immunity during vaccination. J Control Release.

[10] Miyabe H, Hyodo M, Nakamura T, Sato Y, Hayakawa Y, Harashima H (2014). A new adjuvant delivery system 'cyclic di-GMP/YSK05 liposome' for cancer immunotherapy. J Control Release.

[11] Nakamura T, Miyabe H, Hyodo M, Sato Y, Hayakawa Y, Harashima H (2015). Liposomes loaded with a STING pathway ligand, cyclic di-GMP, enhance cancer immunotherapy against metastatic melanoma. J Control Release.

[12] Chen N, Gallovic MD, Tiet P, Ting JPY, Ainslie KM, Bachelder EM (2018). Investigation of tunable acetalated dextran microparticle platform to optimize M2e-based influenza vaccine efficacy. J Control Release.

[13] Cheng N, Watkins-Schulz R, Junkins RD, David CN, Johnson BM, Montgomery SA (2018). A nanoparticle-incorporated STING activator enhances antitumor immunity in PD-L1-insensitive models of triple-negative breast cancer. JCI Insight.

[14] Luo M, Liu Z, Zhang X, Han C, Samandi LZ, Dong C (2019). Synergistic STING activation by PC7A nanovaccine and ionizing radiation improves cancer immunotherapy. J Control Release.

[15] Leach DG, Dharmaraj N, Piotrowski SL, Lopez-Silva TL, Lei YL, Sikora AG (2018). STINGel: controlled release of a cyclic dinucleotide for enhanced cancer immunotherapy. Biomaterials.

[16] Watkins-Schulz R, Tiet P, Gallovic MD, Junkins RD, Batty C, Bachelder EM (2019). A microparticle platform for STING-targeted immunotherapy enhances natural killer cell- and CD8^+^ T cell-mediated anti-tumor immunity. Biomaterials.

[17] Hanson MC, Crespo MP, Abraham W, Moynihan KD, Szeto GL, Chen SH (2015). Nanoparticulate STING agonists are potent lymph node-targeted vaccine adjuvants. J Clin Invest.

[18] Bachelder EM, Beaudette TT, Broaders KE, Dashe J, Fréchet JMJ (2008). Acetal-derivatized dextran: an acid-responsive biodegradable material for therapeutic applications. J Am Chem Soc.

[19] Shae D, Becker KW, Christov P, Yun DS, Lytton-Jean AKR, Sevimli S (2019). Endosomolytic polymersomes increase the activity of cyclic dinucleotide STING agonists to enhance cancer immunotherapy. Nat Nanotechnol.

[20] Nicolai CJ, Wolf N, Chang IC, Kirn G, Marcus A, Ndubaku CO (2020). NK cells mediate clearance of CD8^+^ T cell-resistant tumors in response to STING agonists. Sci Immunol.

[21] Wang-Bishop L, Wehbe M, Shae D, James J, Hacker BC, Garland K (2020). Potent STING activation stimulates immunogenic cell death to enhance antitumor immunity in neuroblastoma. J Immunother Cancer.

[22] Liu Y, Wang L, Song Q, Ali M, Crowe WN, Kucera GL (2022). Intrapleural nano-immunotherapy promotes innate and adaptive immune responses to enhance anti-PD-L1 therapy for malignant pleural effusion. Nat Nanotechnol.

[23] Sun X, Zhang Y, Li J, Park KS, Han K, Zhou X (2021). Amplifying STING activation by cyclic dinucleotide-manganese particles for local and systemic cancer metalloimmunotherapy. Nat Nanotechnol.

[24] Tan X, Jia F, Wang P, Zhang K (2020). Nucleic acid-based drug delivery strategies. J Control Release.

[25] Lächelt U, Wagner E (2015). Nucleic acid therapeutics using polyplexes: a journey of 50 years (and beyond). Chem Rev.

[26] Gupta A, Andresen JL, Manan RS, Langer R (2021). Nucleic acid delivery for therapeutic applications. Adv Drug Deliver Rev.

[27] Whitehead KA, Langer R, Anderson DG (2009). Knocking down barriers: advances in siRNA delivery. Nat Rev Drug Discov.

[28] Ma Y, Zhu Y, Wang C, Pan D, Liu S, Yang M (2018). Annealing novel nucleobase-lipids with oligonucleotides or plasmid DNA based on H-bonding or π-π interaction: assemblies and transfections. Biomaterials.

[29] Ma X, Sun J, Qiu C, Wu Y, Zheng Y, Yu M (2016). The role of disulfide-bridge on the activities of H-shape gemini-like cationic lipid based siRNA delivery. J Control Release.

[30] Ma Y, Zhao W, Li Y, Pan Y, Wang S, Zhu Y (2019). Structural optimization and additional targets identification of antisense oligonucleotide G3139 encapsulated in a neutral cytidinyl-lipid combined with a cationic lipid *in vitro* and in vivo. Biomaterials.

[31] Zhang Y, Li S, Zhou X, Sun J, Fan X, Guan Z (2019). Construction of targeting nanoparticle of 3',3"-bis-peptide-siRNA conjugate/mixed lipid with post-inserted DSPE-PEG2000-cRGD. Mol Pharmaceut.

[32] Zhou X, Pan Y, Li Z, Li H, Wu J, Ma Y (2020). siRNA packaged with neutral cytidinyl/cationic/PEG lipids for enhanced antitumor efficiency and safety *in vitro* and in vivo. ACS Appl Bio Mater.

[33] Zhou X, Pan Y, Yu L, Wu J, Li Z, Li H (2021). Feasibility of cRGD conjugation at 5′-antisense strand of siRNA by phosphodiester linkage extension. Mol Ther-Nucleic Acids.

[34] Li L, Long J, Sang Y, Wang X, Zhou X, Pan Y (2021). Rational preparation and application of a mRNA delivery system with cytidinyl/cationic lipid. J Control Release.

[35] Guan J, Pan Y, Li H, Zhu Y, Gao Y, Wang J Activity and tissue distribution of antisense oligonucleotide CT102 encapsulated with cytidinyl/cationic lipid against hepatocellular carcinoma. 2022; [Epub ahead of print].

[36] McLane LM, Abdel-Hakeem MS, Wherry EJ (2019). CD8 T cell exhaustion during chronic viral infection and cancer. Annu Rev Immunol.

[37] Qi N, Jung K, Wang M, Na LX, Yang ZJ, Zhang LR (2011). A novel membrane-permeant cADPR antagonist modified in the pyrophosphate bridge. Chem Commun.

[38] Dai H, Yu XT, Guan Z, Zhang LH, Yang ZJ (2019). Synthesis of c-di-GMP analogs modified by ganciclovir and biological activity to induce type I interferon. Chin J Pharm Sci.

[39] Doshi AS, Cantin S, Prickett LB, Mele DA, Amiji M (2022). Systemic nano-delivery of low-dose STING agonist targeted to CD103^+^ dendritic cells for cancer immunotherapy. J Control Release.

[40] Faustino-Rocha A, Oliveira PA, Pinho-Oliveira J, Teixeira-Guedes C, Soares-Maia R, Da Costa RG (2013). Estimation of rat mammary tumor volume using caliper and ultrasonography measurements. Lab Animal.

[41] Allen BM, Hiam KJ, Burnett CE, Venida A, DeBarge R, Tenvooren I (2020). Systemic dysfunction and plasticity of the immune macroenvironment in cancer models. Nat Med.

[42] Fischer AH, Jacobson KA, Rose J, Zeller R Hematoxylin and eosin staining of tissue and cell sections. Cold Spring Harb. Protoc. 2008; 3, pdb.prot4986.

[43] Cai X, Chiu YH, Chen ZJ (2014). The cGAS-cGAMP-STING pathway of cytosolic DNA sensing and signaling. Mol Cell.

[44] Abe T, Barber GN (2014). Cytosolic-DNA-mediated, STING-dependent proinflammatory gene induction necessitates canonical NF-kB activation through TBK1. J Virol.

[45] Parker BS, Rautela J, Hertzog PJ (2016). Antitumour actions of interferons: implications for cancer therapy. Nat Rev Cancer.

[46] Gardner A, Ruffell B (2016). Dendritic cells and cancer immunity. Trends Immunol.

[47] Neophytou C, Boutsikos P, Papageorgis P (2018). Molecular mechanisms and emerging therapeutic targets of triple-negative breast cancer metastasis. Front Oncol.

[48] Boxberg M, Leising L, Steiger K, Jesinghaus M, Alkhamas A, Mielke M (2018). Composition and clinical impact of the immunologic tumor microenvironment in oral squamous cell carcinoma. J Immunol.

[49] Sato E, Olson SH, Ahn J, Bundy B, Nishikawa H, Qian F (2005). Intraepithelial CD8^+^ tumor-infiltrating lymphocytes and a high CD8^+^/regulatory T cell ratio are associated with favorable prognosis in ovarian cancer. Proc Natl Acad Sci USA.

[50] Ries CH, Cannarile MA, Hoves S, Benz J, Wartha K, Runza V (2014). Targeting tumor-associated macrophages with anti-CSF-1R antibody reveals a strategy for cancer therapy. Cancer Cell.

[51] Mazzaschi G, Madeddu D, Falco A, Bocchialini G, Goldoni M, Sogni F (2018). Low PD-1 expression in cytotoxic CD8^+^ tumor-infiltrating lymphocytes confers an immune-privileged tissue microenvironment in NSCLC with a prognostic and predictive value. Clin Cancer Res.

[52] Nagarsheth N, Wicha MS, Zou W (2017). Chemokines in the cancer microenvironment and their relevance in cancer immunotherapy. Nat Rev Immunol.

[53] Philip M, Fairchild L, Sun L, Horste EL, Camara S, Shakiba M (2017). Chromatin states define tumour-specific T cell dysfunction and reprogramming. Nature.

[54] Chen J, López-Moyado IF, Seo H, Lio CJ, Hempleman LJ, Sekiya T (2019). NR4A transcription factors limit CAR T cell function in solid tumours. Nature.

[55] Seo H, Chen J, González-Avalos E, Samaniego-Castruita D, Das A, Wang YH (2019). TOX and TOX2 transcription factors cooperate with NR4A transcription factors to impose CD8^+^ T cell exhaustion. Proc Natl Acad Sci USA.

