# Supplementary material for: Novel formulation of c-di-GMP with cytidinyl/cationic lipid reverses T cell exhaustion and activates stronger anti-tumor immunity
Source: Theranostics. 2022 Sep 11;12(15):6723–39. doi: 10.7150/thno.71010 (PMC9516232; doi:10.7150/thno.71010)
Supplement: Supplementary file 1 — Supplementary figures and tables. [file thnov12p6723s1.pdf]

# Supplementary Information

# Novel formulation of c-di-GMP with cytidinyl/cationic lipid reverses T cell exhaustion and activates stronger anti-tumor immunity

Xiaotong Yu[1*], Jing Yu[1*], Hong Dai[1], Chenyun Deng[1], Xudong Sun[1], Sijie Long[1], Zhujun Jiang[1], Hongyan Jin[2], Zhu Guan[1], Zhenjun Yang[1]✉

[1]State Key Laboratory of Natural & Biomimetic Drugs, School of Pharmaceutical Sciences, Peking University, No.38 Xueyuan Road, Haidian District, Beijing 100191, China.

[2]Department of Obstetrics and Gynecology, Peking University First Hospital, No. 8 Xishiku Street, 100034, Beijing, China.

[*] Authors contributed equally to this work.

✉ Corresponding author: E-mail: yangzj@bjmu.edu.cn(Z. Yang)

**Table S1**. Primers of several mRNAs used in this study

| Gene symbols | Types/primer | Sequence (5′-3′) |
|---|---|---|
| *Ifnβ* | Forward | GCCTTTGCCATCCAAGAGATGC |
| | Reverse | ACACTGTCTGCTGGTGGAGTTC |
| *Il-6* | Forward | TACCACTTCACAAGTCGGAGGC |
| | Reverse | CTGCAAGTGCATCATCGTTGTTC |
| *Cxcl9* | Forward | CCTAGTGATAAGGAATGCACGATG |
| | Reverse | CTAGGCAGGTTTGATCTCCGTTC |
| *Cxcl10* | Forward | ATCATCCCTGCGAGCCTATCCT |
| | Reverse | GACCTTTTTTGGCTAAACGCTTTC |
| *Tox* | Forward | AGTCACCCAGTCGTCTCTT |
| | Reverse | TTCTCCTCTCTCTCCTTCATCTC |
| *Nr4a* | Forward | TGCGTGCAAGCCCAGTATAG |
| | Reverse | ATAAGTCTGCGTGGCGTAAGT |

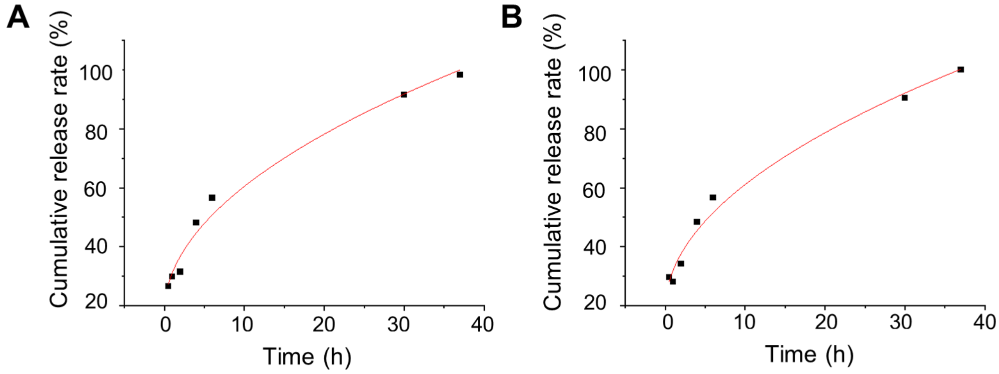


**Figure S1.** Cumulative-release profile of cdG from DNCA/CLD/cdG **(A)** and DNCA/CLD/Ca/cdG **(B)** incubated with PBS at 37 °C (n = 3).

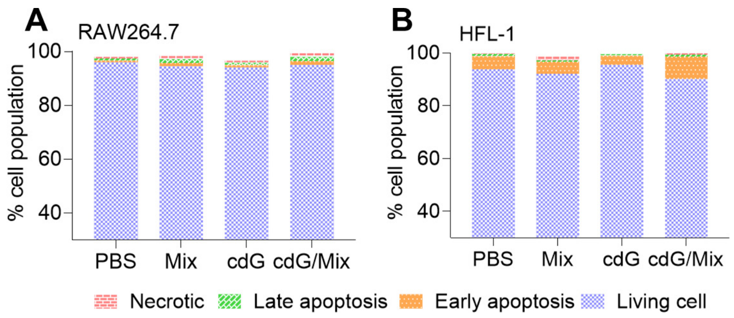


**Figure S2.** Flow cytometric analysis of the cell apoptosis induced by indicated formulations in RAW264.7 **(A)** and HFL-1 **(B)**.

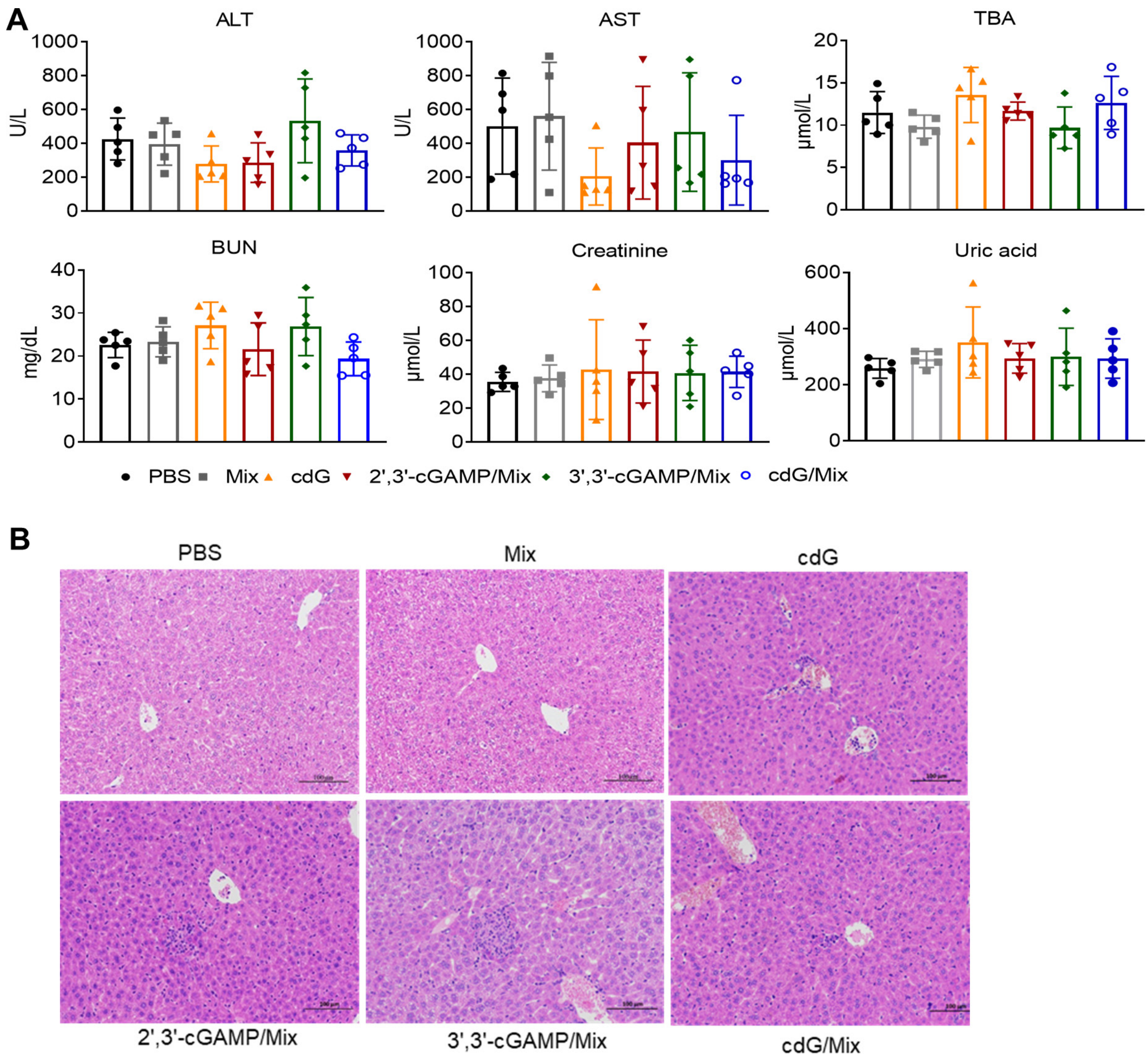


**Figure S3. (A)** Blood levels of alanine aminotransferase (ALT), aspartate aminotransferase (AST), total bile acid (TBA), blood urea nitrogen (BUN), uric acid and creatinine. Blood was harvested when mice reached tumor size endpoints. Data are presented as mean ± SD (n=5). **(B)** Representative images H&E stained liver sections harvested on reaching the tumor size endpoint. No significant pathological changes were observed in the liver between control and treatment group (n=3).

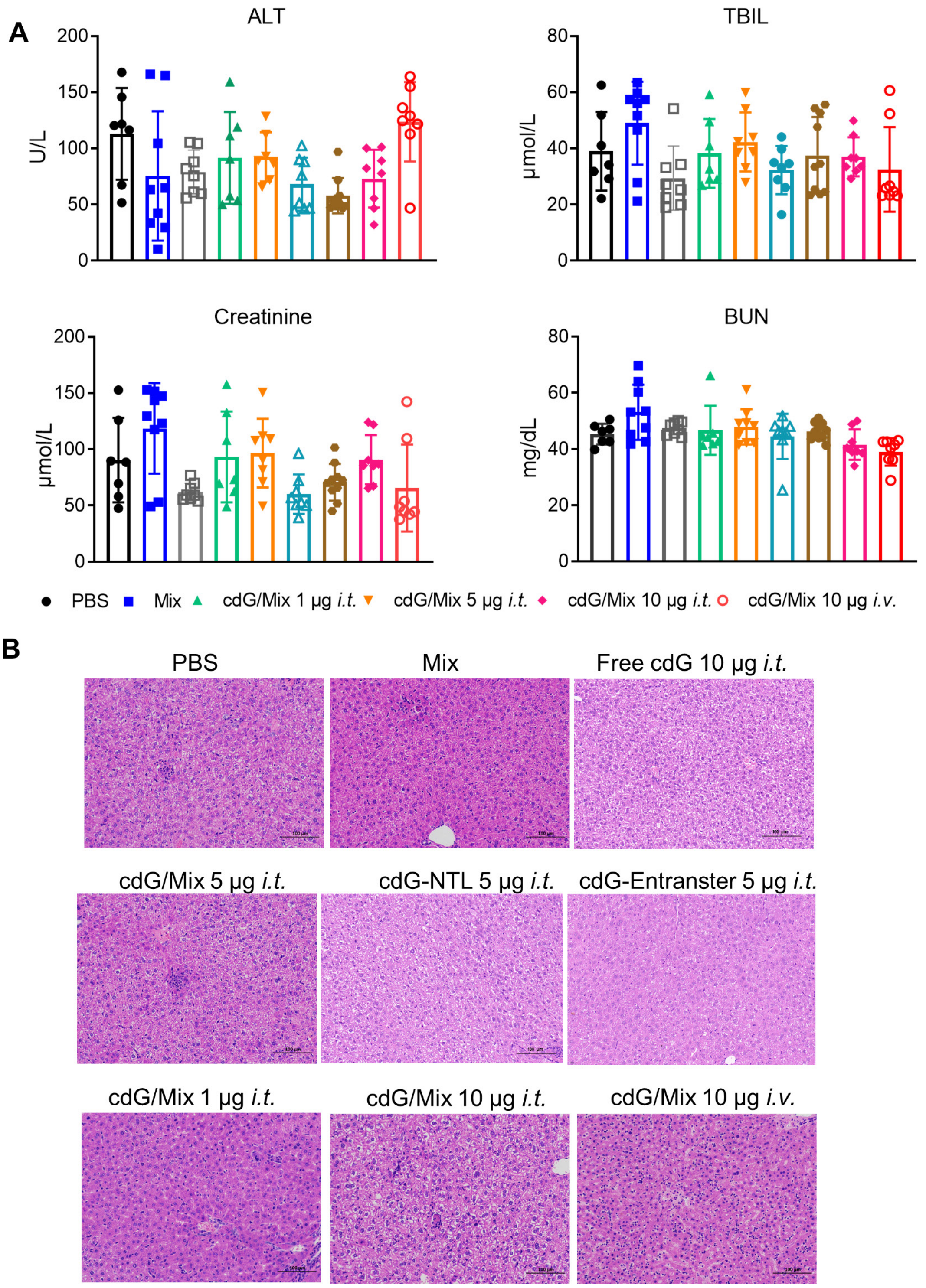


**Figure S4. (A)** Blood levels of alanine aminotransferase (ALT), total bilirubin (TBIL), creatinine and blood urea nitrogen (BUN), Blood was harvested when mice reached tumor size endpoints. Data are presented as mean ± SD (n=7-9). **(B)** Representative images H&E stained liver sections harvested on reaching the tumor size endpoint. No significant pathological changes were observed in the liver between control and treatment group (n=3).

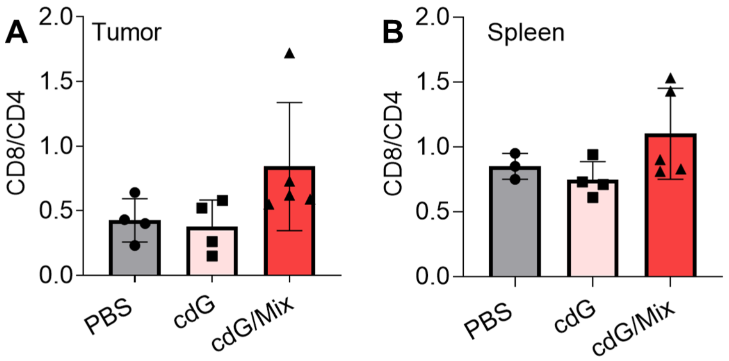


**Figure S5.** Ratio of CD8$^{+}$ to CD4$^{+}$ T cells in the TME(A) and spleen(B) (n = 3-5; one-way ANOVA). cdG(5 μg) and cdG(5μg)/Mix.

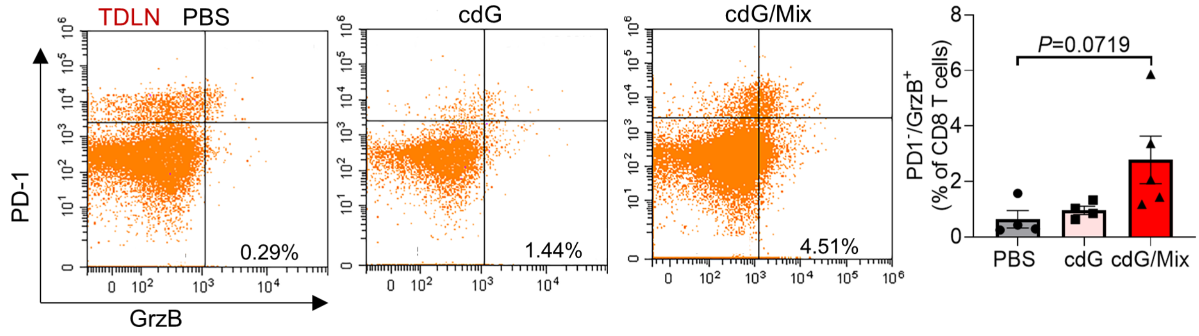


**Figure S6.** Representative scatter plots and quantification of the proportion of Granzyme B$^{+}$ PD-1$^{-}$ CD8$^{+}$ T cells in TDLN of EO771-tumor-bearing mice 48 h after single dose of indicated formulation by intratumoral administration. All panels are from one experiment (n = 4-5; one-way ANOVA). cdG(5 μg) and cdG(5 μg)/Mix.

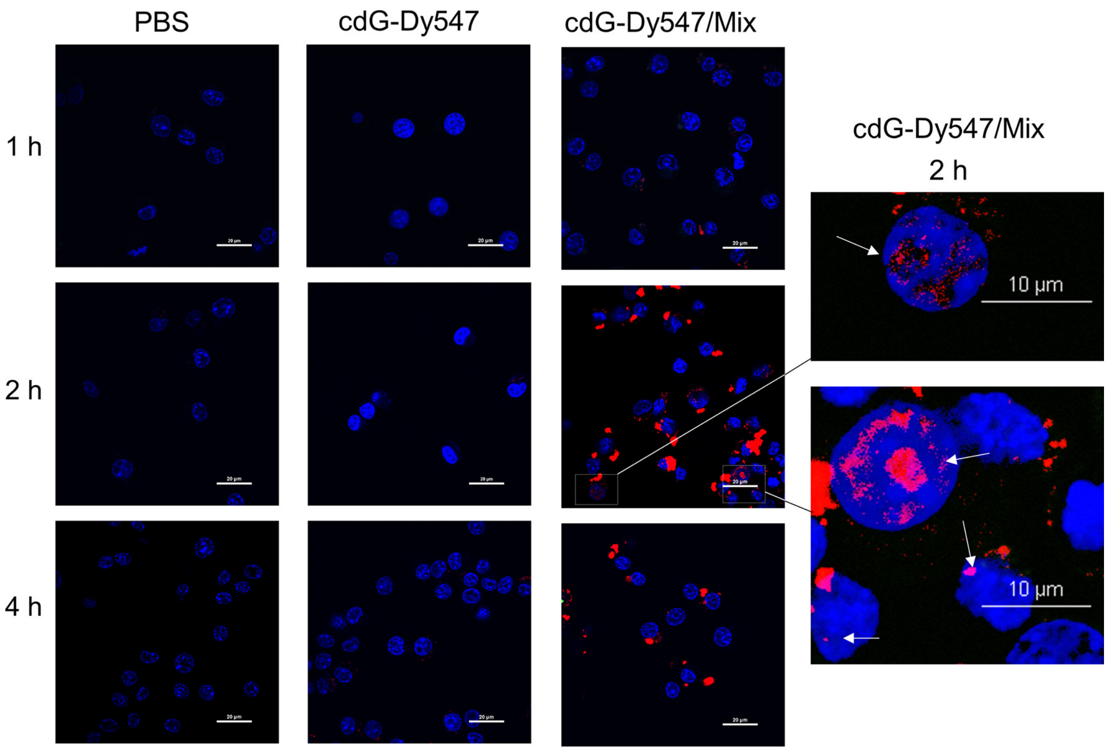


**Figure S7.** Confocal microscopy time course (scale bar: 20 μm) and magnified images (scale bar: 10 μm) of uptake and intracellular distribution of cdG-Dy547 in RAW264.7 cells Arrows indicate representative co-localization of nucleus and cdG-Dy547. Representative images from three slides/condition.

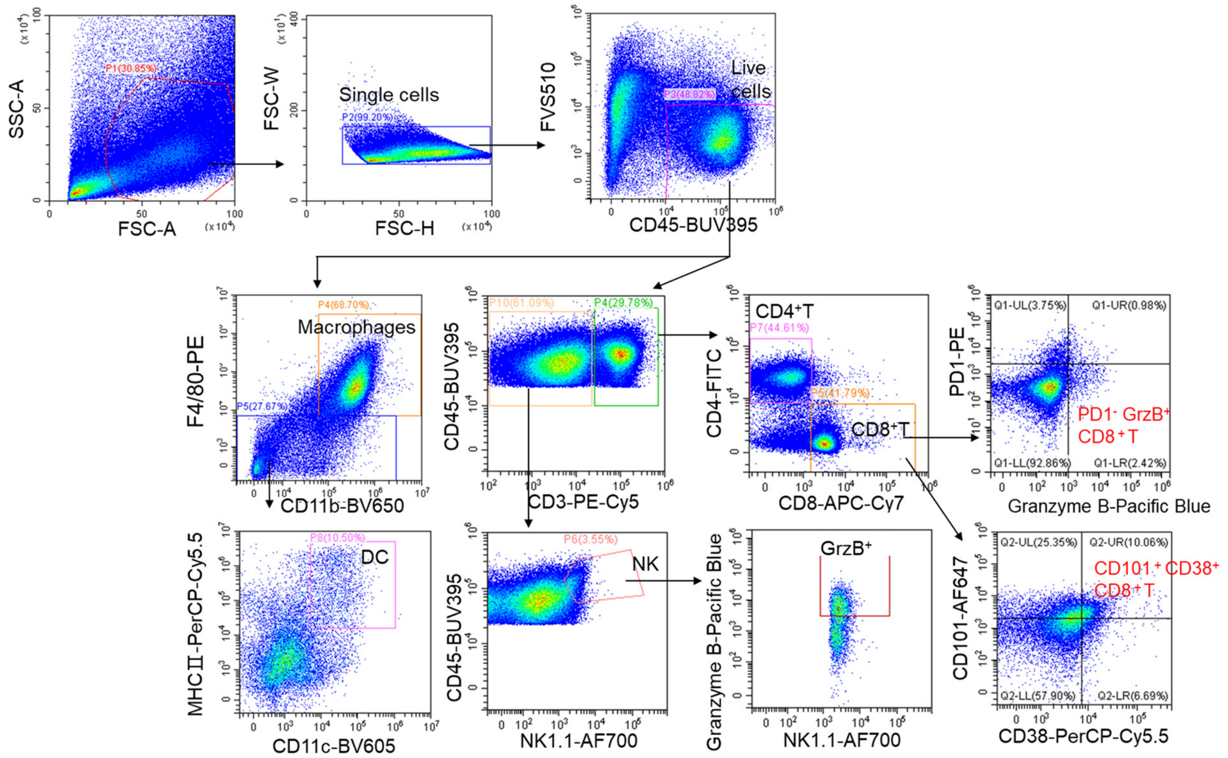

**Figure S8.** Flow cytometry gating strategy of Figure 7-9 and 11.

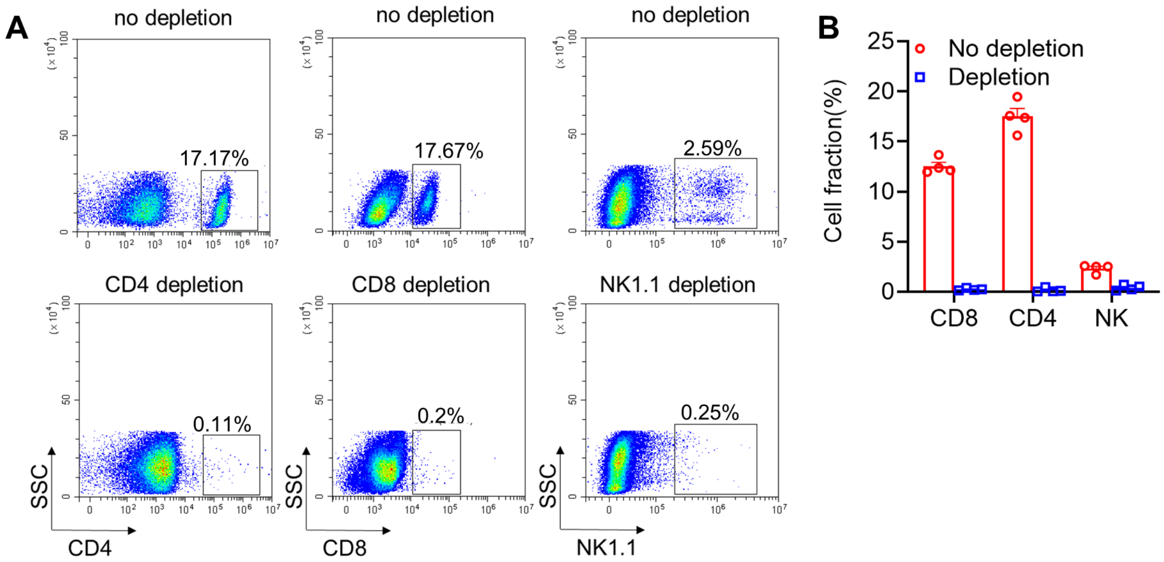


**Figure S9.** Specific cellular subsets depletions confirmed in blood. Representative scatter plots **(A)** and quantification **(B)** of the proportion of indicated cellular subsets. Four mice were randomly selected per group for flow cytometry.

**General Information.** Anhydrous solvents (acetovnitrile, with water ≤10 ppm) were purchased from 3A Chemical (Shanghai) Technology Co., Ltd. The phosphoramidite reagents were purchased from Wuhu Huaren Science and Technology Co., Ltd. without further purification. Reactions were checked with TLC (Merck precoated 60F254 plates). Column chromatography were performed on silica gel 60 (200–300 mesh, Merck) using gradients of $CH_3OH/CH_2Cl_2$. The compounds purification was performed on Venusil XBP preparative $C_{18}$ reversed-phase column (10 μm, 21.2 × 250 mm) on Gilson HPLC with MeCN/TEAB as mobile phase. $^1$H NMR and $^{31}$P NMR spectra were recorded on Bruker Avance III 400 at room temperature and were reported in ppm. Mass spectra (ESI-TOF) were recorded on MDS SCIEX QSTAR High performance liquid chromatography-mass spectrometry; HR-ESI-MS spectra were recorded on Bruker APEX IV mass spectrometry.

**General procedures for cyclic dinucleotides synthesis.** All of the analogues concerning cyclic dinucleotides reported in this paper were synthesized by the phosphoramidite method developed by our group (**Scheme 1**). The synthetic procedures for different kinds of analogue are basically the same except for some changes in the substrates. Taking c-di-GMP (**5a)** as example, compound **1** (1 eq.), compound **2** (1 eq.) and 1*H*-tetrazole (3 eq.) were dissolved in

anhydrous acetonitrile under argon. The reaction was stirred at room temperature for 5 h and then was oxidized by *tert*-butyl hydroperoxide (TBHP) in decane. After detritylation with trifluoroacetic acid, coupling product **3a** was obtained which was further cyclized using $P^{III}$ agent **6** catalyzed by 1*H*-tetrazole. And the cyclization product was then oxidized via TBHP, deprotected via methylamine/methanol and $Et_3N{\cdot}3HF$. The final product **5a** was collected after HPLC purification.

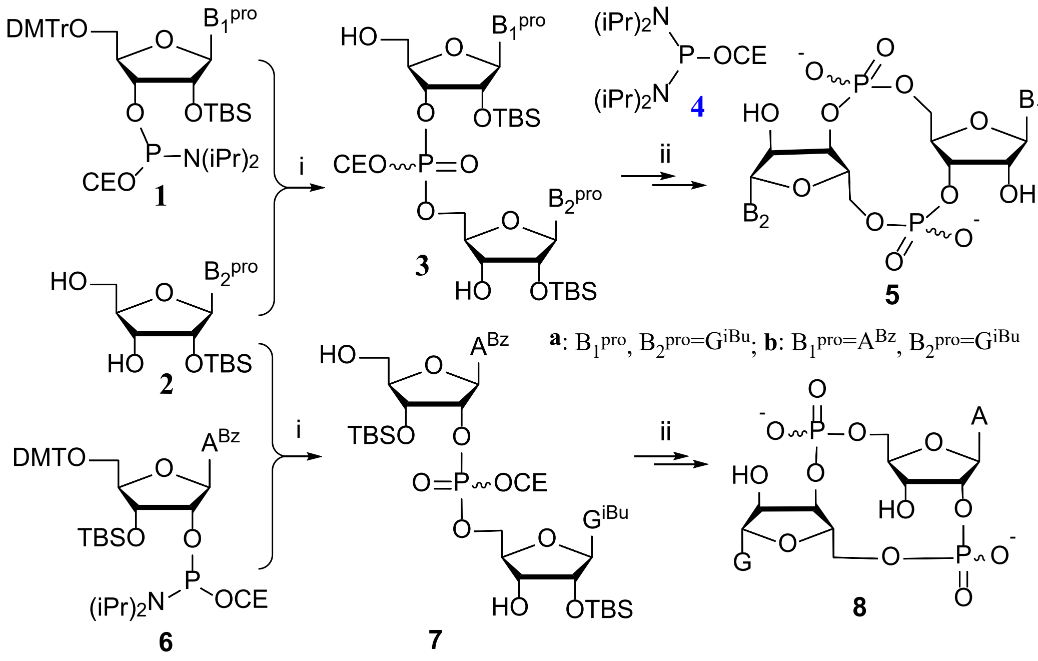


**Scheme 1.** The reported one-pot phosphoramidite method for the synthesis of cyclic dinucleotides optimized by our group. Reagents and conditions: i. (a) 1*H*-tetrazole, anhyhrous $CH_3CN$, rt, 5 h; (b) TBHP (5-6 M in decane), rt, 10 min or $S_8$, rt, 12 h; (c) $CF_3COOH$, $CH_2Cl_2$, rt, 20 min; ii. (a) **4**, 1*H*-tetrazole, anhyhrous $CH_3CN$, rt, 5 h; (b) TBHP (5-6 M in decane), rt, 10 min or $S_8$, rt, 12 h; (c) $CH_3NH_2$ in MeOH, rt, 3 h; (d) $Et_3N{\cdot}3HF$, pyridine, 50 °C, 1 h;

*Data for* ***5a****(c-di-GMP) (exist as triethylammonium salt)*. $^1$H NMR (400 MHz, $D_2O$) $\delta$ 7.91 (s, 2H, 8-H), 5.83 (s, 2H, 1′-H), 4.82 (m, 2H), 4.64 (d, $J$ = 4.5 Hz, 2H), 4.27 (m, 4H), 3.98 (dd, $J$ = 11.4, 3.4 Hz, 2H). $^{31}$P NMR (162 MHz, $D_2O$) $\delta$ -1.43 (s). HRMS (ESI-TOF$^-$) Calcd for $C_{20}H_{23}N_{10}O_{14}P_2$ [(M-H)$^-$]: 689.0876, Found 689.0879.

*Data for* ***5b****(3',3'-cGAMP) (exist as triethylammonium salt)*. $^1$H NMR (400 MHz, $D_2O$) $\delta$ 8.20 (s, 1H, 2-H), 8.15 (s, 1H, 8-H), 7.75 (s, 1H, 8″-H), 6.06 (s, 1H), 5.83 (d, $J$ = 8.5 Hz, 1H), 5.54 (td, $J$ = 8.0, 4.1 Hz, 1H), 4.94 (dt, $J$ = 10.3, 5.9 Hz, 1H), 4.48 (d, $J$ = 4.2 Hz, 1H), 4.41-4.26

(m, 4H), 4.17-4.08 (m, 1H), 4.07 -3.99 (m, 2H), 3.07 [q, *J* = 7.3 Hz, 14H, $(CH_3CH_2)_3N$], 1.14 [t, *J* = 7.3 Hz, 18H, $(CH_3CH_2)_3N$]. $^{31}$P NMR (162 MHz, $D_2O$) $\delta$ -1.35, -2.38. HRMS (ESI-TOF$^-$) Calcd for $C_{20}H_{23}N_{10}O_{13}P_2$ [(M-H)$^-$]: 673.0693, Found 673.0699

*Data for **8** (2',3'-cGAMP) (exist as triethylammonium salt).* $^1$H NMR (400 MHz, $D_2O$) $\delta$ 8.15 (s, 1H), 7.86 (s, 1H), 7.77 (s, 1H), 5.88 (s, 1H), 5.70 (s, 1H), 4.99-4.87 (m, 2H), 4.77 (d, *J* = 5.0 Hz, 1H), 4.72 (d, *J* = 3.0 Hz, 1H), 4.39-4.23 (m, 4H), 3.97 (td, *J* = 12.7, 4.3 Hz, 2H). $^{31}$P NMR (162 MHz, $D_2O$) $\delta$ -1.62, -1.74. HRMS (ESI-TOF$^-$) Calcd for $C_{20}H_{23}N_{10}O_{13}P_2$ [(M-H)$^-$]: 673.0921; Found: 673.0922.

## $^1$H, $^{31}$P NMR and HRMS Spectra

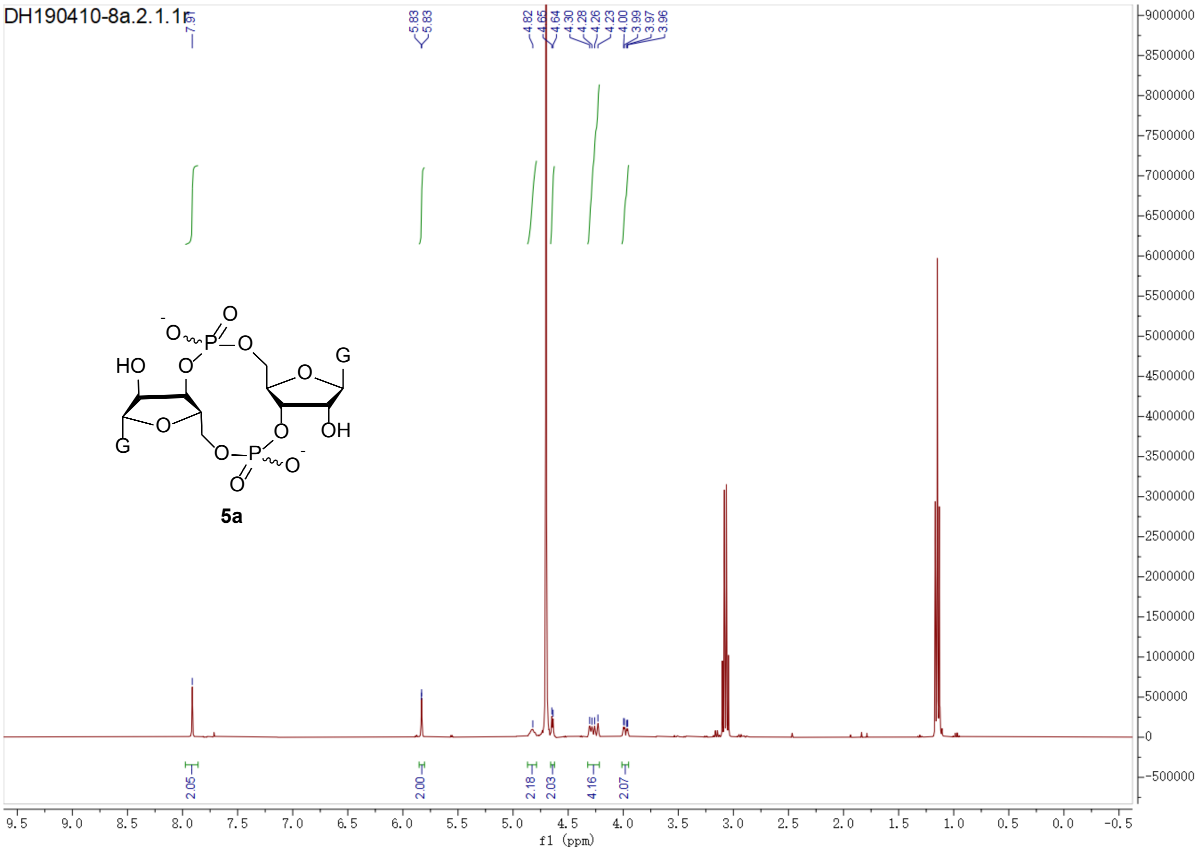


**Figure S10.** $^1$H NMR spectrum of **5a**

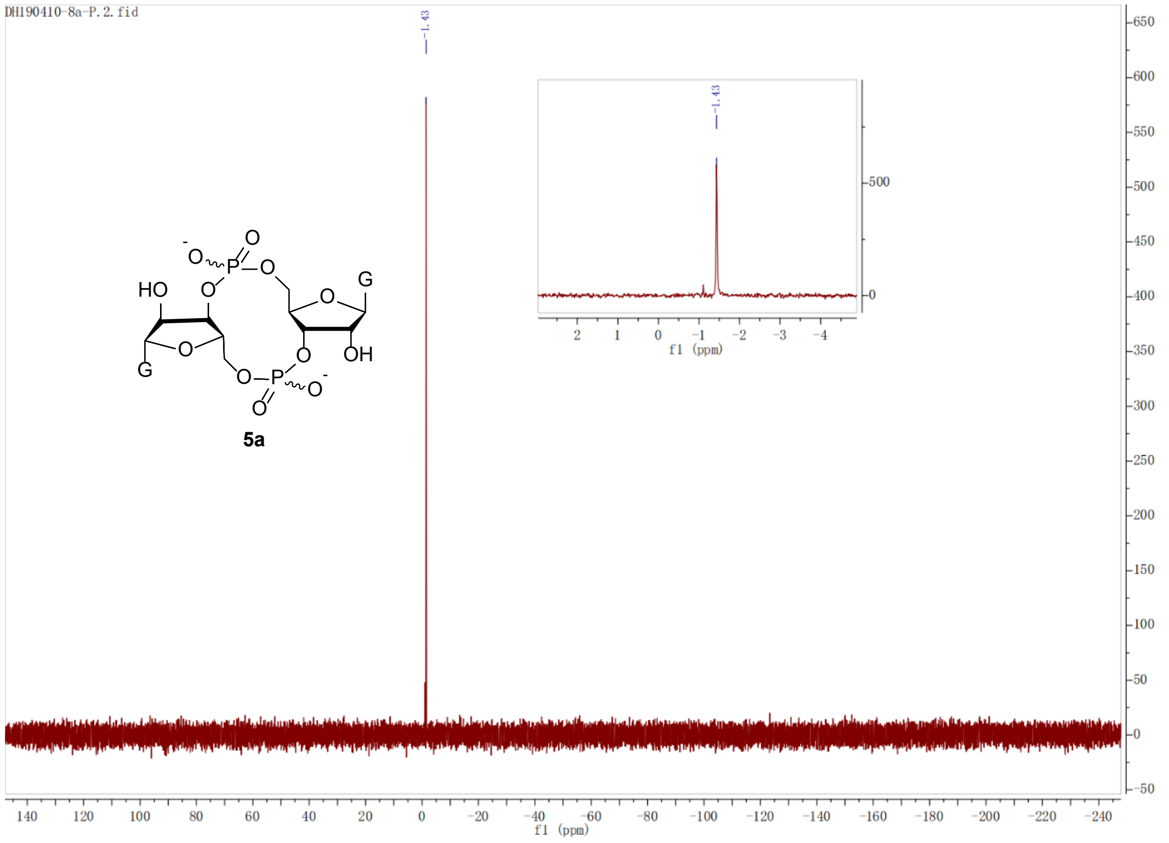


**Figure S11.** $^{31}$P NMR spectrum of **5a**

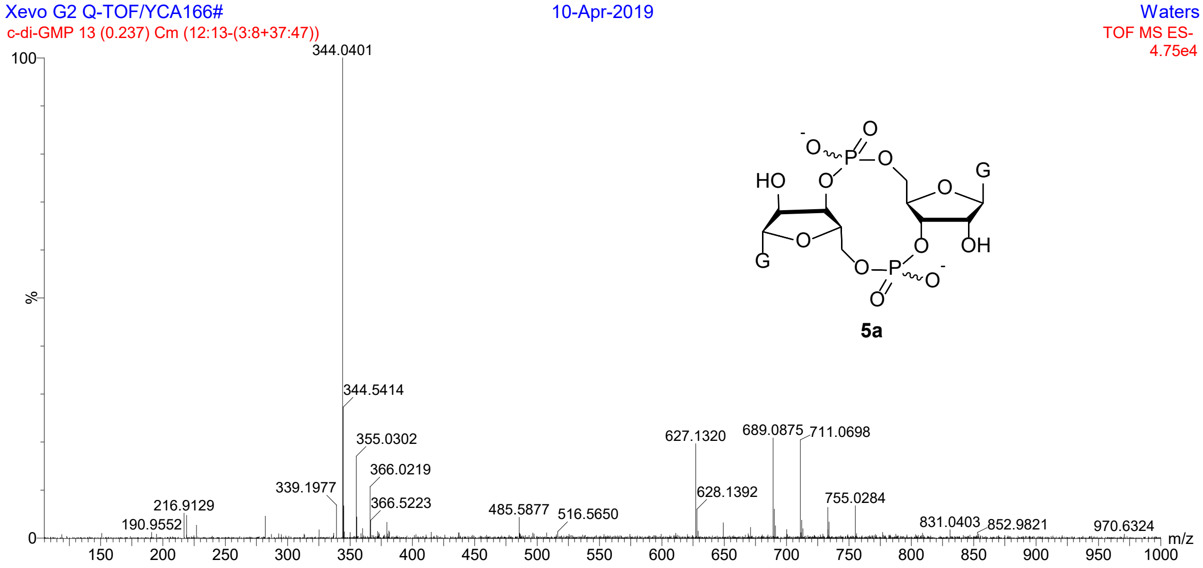


**Figure S12.** HRMS spectrum of **5a**

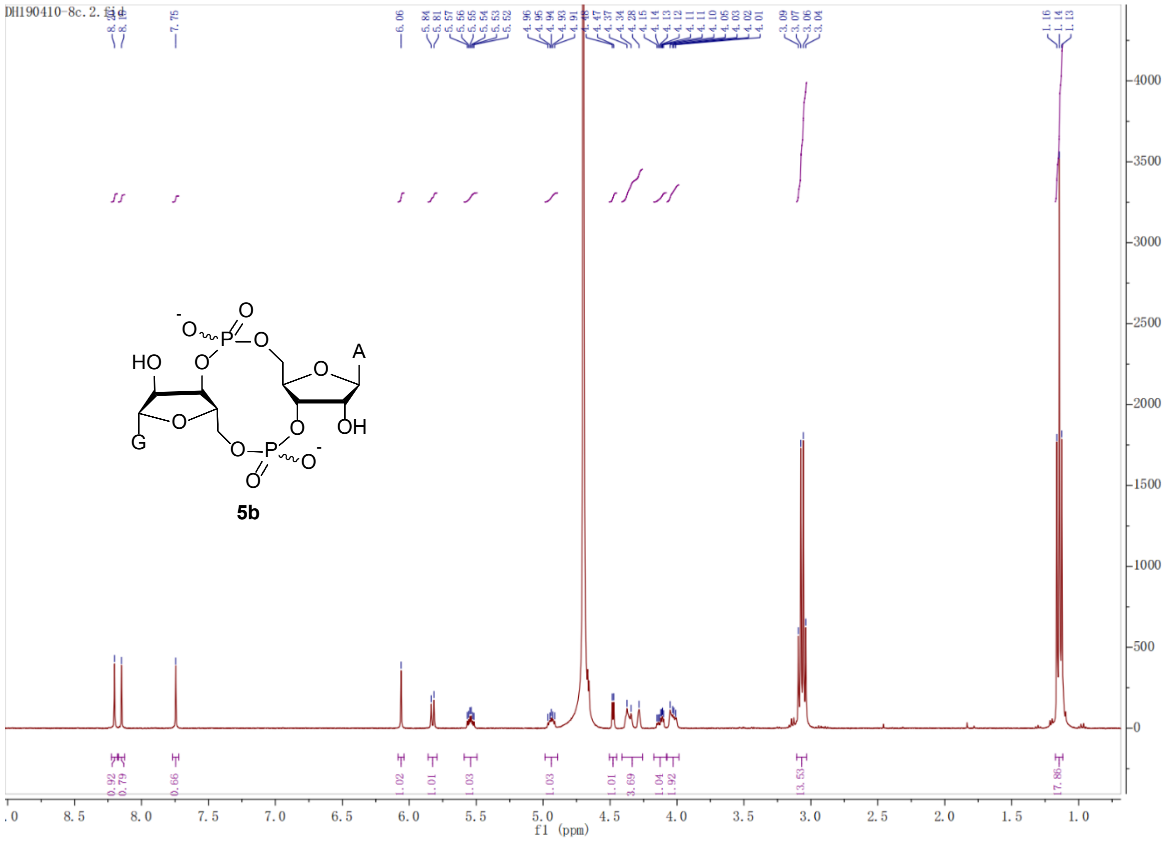

**Figure S13.** $^{1}$H NMR spectrum of **5b**

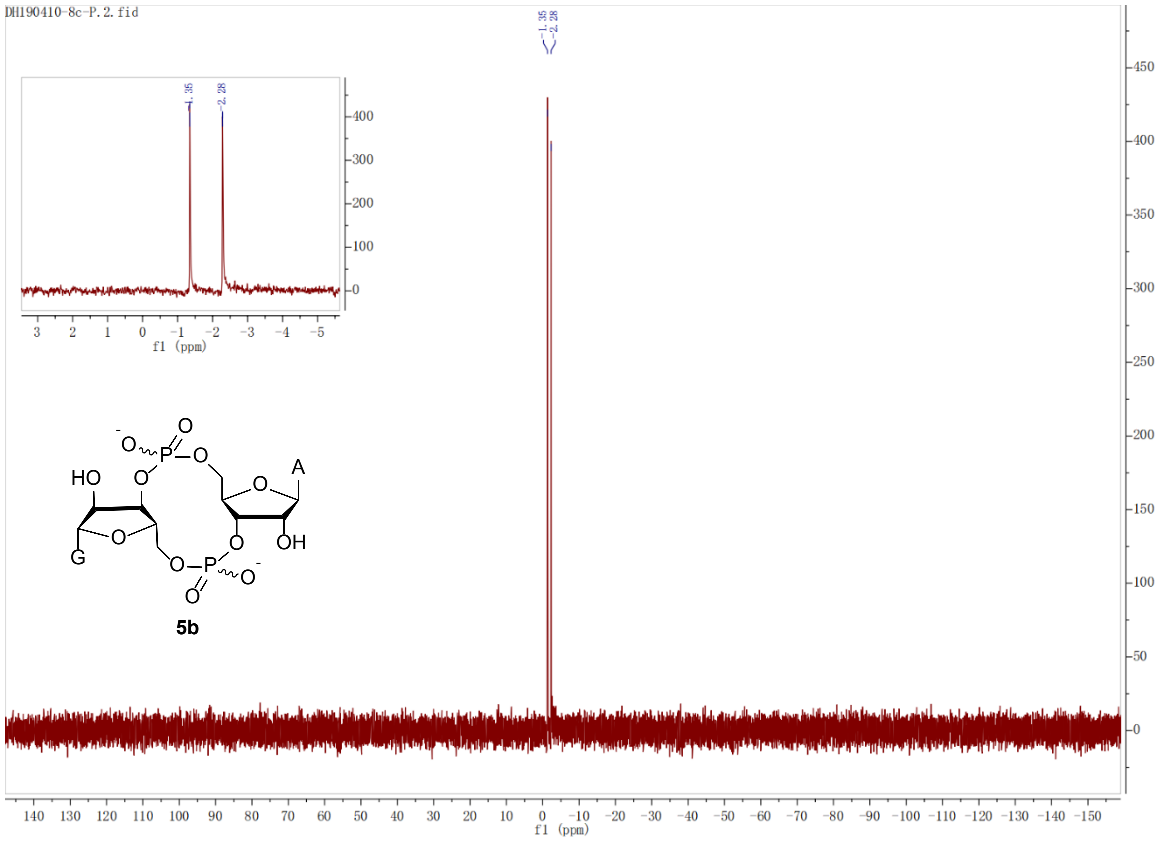

**Figure S14.** $^{31}$P NMR spectrum of **5b**

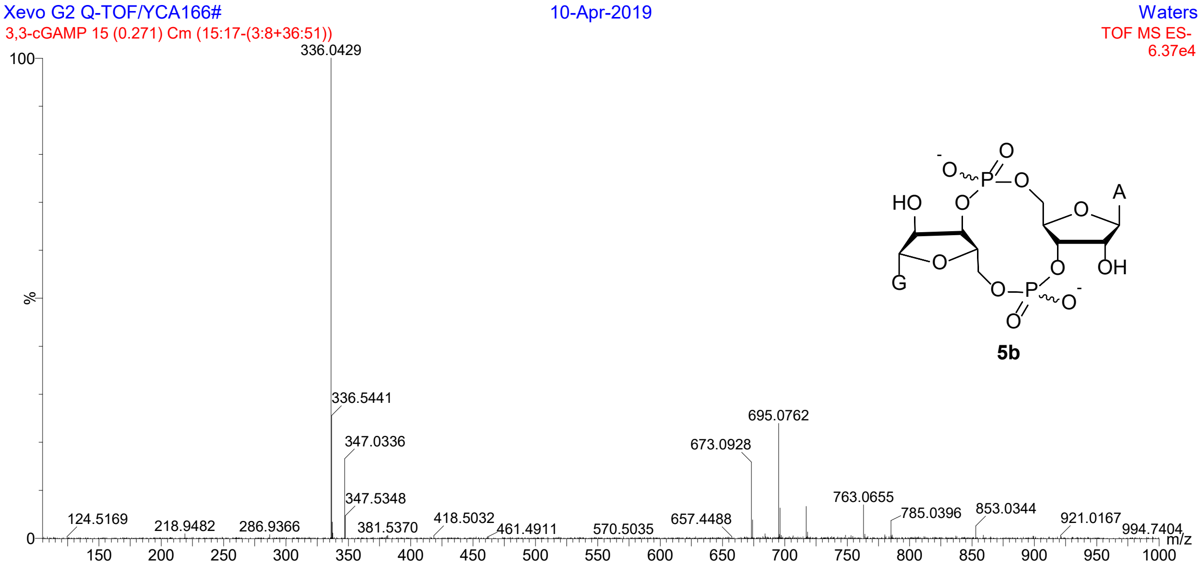


**Figure S15.** HRMS spectrum of **5b**

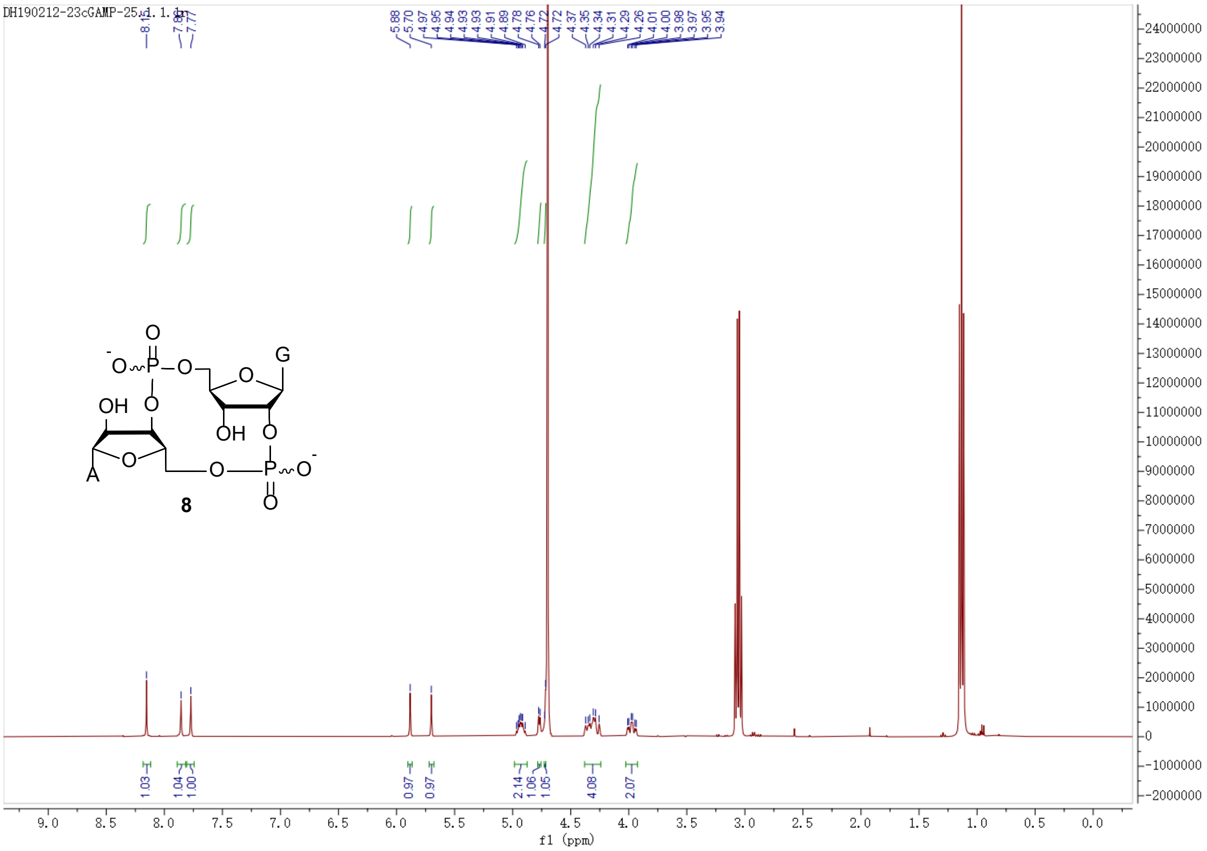


**Figure S16.** $^{1}$H NMR spectrum of **8**

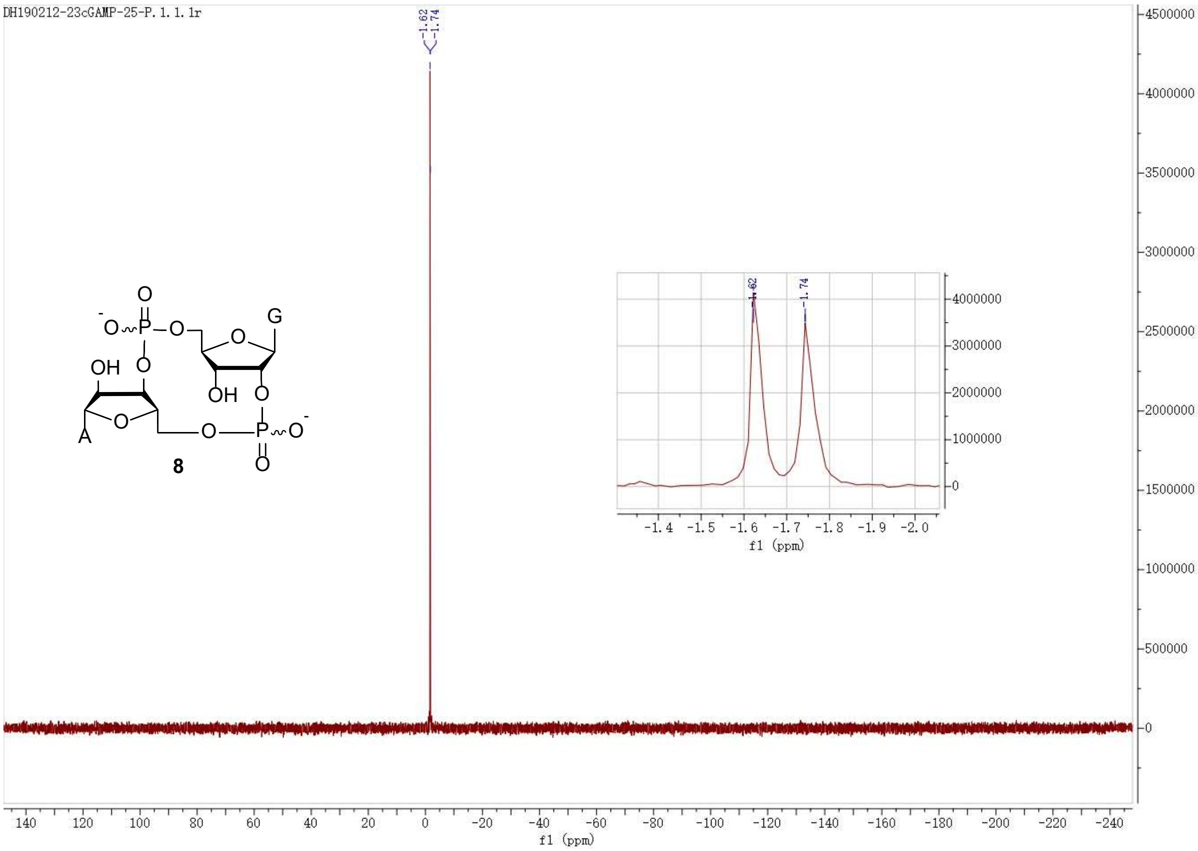


**Figure S17.** $^{31}$P NMR spectrum of **8**

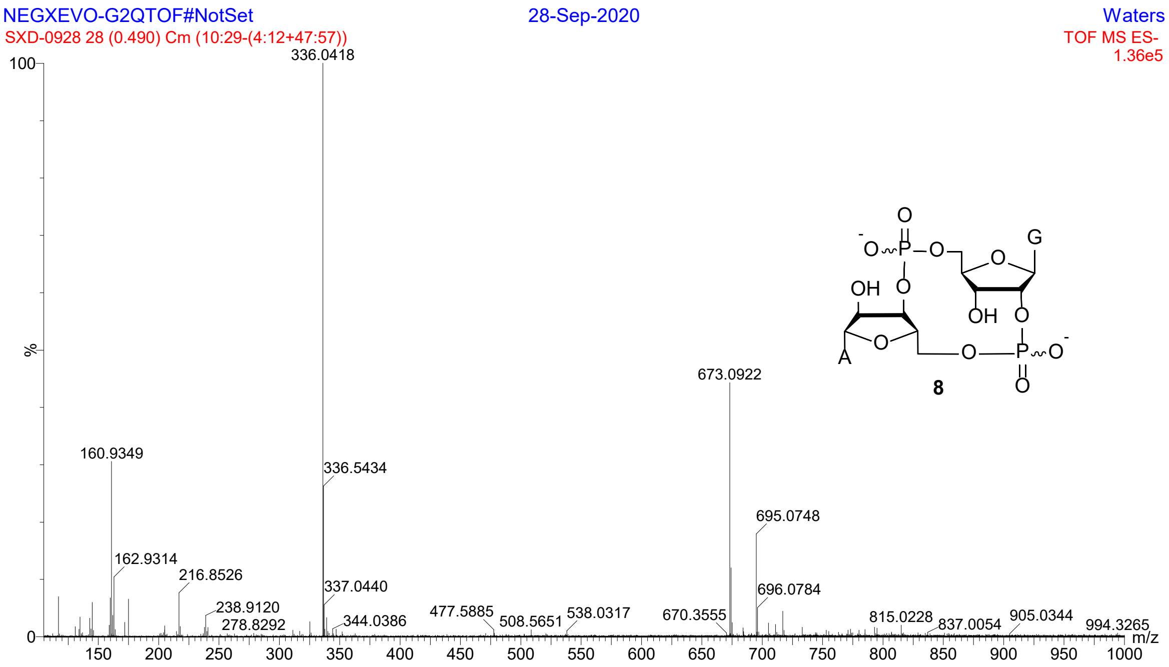


**Figure S18.** HRMS spectrum of **8**
